# Microglia modulate blood flow, neurovascular coupling, and hypoperfusion via purinergic actions

**DOI:** 10.1084/jem.20211071

**Published:** 2022-02-24

**Authors:** Eszter Császár, Nikolett Lénárt, Csaba Cserép, Zsuzsanna Környei, Rebeka Fekete, Balázs Pósfai, Diána Balázsfi, Balázs Hangya, Anett D. Schwarcz, Eszter Szabadits, Dávid Szöllősi, Krisztián Szigeti, Domokos Máthé, Brian L. West, Katalin Sviatkó, Ana Rita Brás, Jean-Charles Mariani, Andrea Kliewer, Zsolt Lenkei, László Hricisák, Zoltán Benyó, Mária Baranyi, Beáta Sperlágh, Ákos Menyhárt, Eszter Farkas, Ádám Dénes

**Affiliations:** 1 “Momentum” Laboratory of Neuroimmunology, Institute of Experimental Medicine, Budapest, Hungary; 2 János Szentágothai Doctoral School of Neurosciences, Schools of PhD Studies, Semmelweis University, Budapest, Hungary; 3 Lendület Laboratory of Systems Neuroscience, Institute of Experimental Medicine, Budapest, Hungary; 4 Department of Biophysics and Radiation Biology, Semmelweis University, Budapest, Hungary; 5 Hungarian Centre of Excellence for Molecular Medicine, Szeged, Hungary; 6 Plexxikon Inc., Berkeley, CA; 7 Institute of Psychiatry and Neurosciences of Paris, INSERM U1266, Université de Paris, Paris, France; 8 Institute of Translational Medicine, Semmelweis University, Budapest, Hungary; 9 Laboratory of Molecular Pharmacology, Institute of Experimental Medicine, Budapest, Hungary; 10 Hungarian Centre of Excellence for Molecular Medicine, University of Szeged, Cerebral Blood Flow and Metabolism Research Group, Szeged, Hungary; 11 Department of Medical Physics and Informatics, Albert Szent-Györgyi Medical School, University of Szeged, Szeged, Hungary; 12 Department of Cell Biology and Molecular Medicine, Albert Szent-Györgyi Medical School, Faculty of Science and Informatics, University of Szeged, Szeged, Hungary

## Abstract

Microglia, the main immunocompetent cells of the brain, regulate neuronal function, but their contribution to cerebral blood flow (CBF) regulation has remained elusive. Here, we identify microglia as important modulators of CBF both under physiological conditions and during hypoperfusion. Microglia establish direct, dynamic purinergic contacts with cells in the neurovascular unit that shape CBF in both mice and humans. Surprisingly, the absence of microglia or blockade of microglial P2Y12 receptor (P2Y12R) substantially impairs neurovascular coupling in mice, which is reiterated by chemogenetically induced microglial dysfunction associated with impaired ATP sensitivity. Hypercapnia induces rapid microglial calcium changes, P2Y12R-mediated formation of perivascular phylopodia, and microglial adenosine production, while depletion of microglia reduces brain pH and impairs hypercapnia-induced vasodilation. Microglial actions modulate vascular cyclic GMP levels but are partially independent of nitric oxide. Finally, microglial dysfunction markedly impairs P2Y12R-mediated cerebrovascular adaptation to common carotid artery occlusion resulting in hypoperfusion. Thus, our data reveal a previously unrecognized role for microglia in CBF regulation, with broad implications for common neurological diseases.

## Teaser

Microglia modulate cerebral blood flow (CBF) through complex purinergic actions, which are mediated by dynamic, P2Y12 receptor-driven interactions with the vasculature. Microglial actions are partially independent of nitric oxide. Microglial dysfunction leads to impaired CBF response during neurovascular coupling, hypercapnia-induced vasodilation and cerebral hypoperfusion.

## Introduction

Microglia are key regulators of inflammatory processes in the brain and altered microglial activity is linked to the development of common brain diseases (Colonna and Butovsky, 2017; Prinz and Priller, 2014). The contribution of microglia to diverse physiological processes, including brain development, synaptic plasticity, learning, or memory is also emerging (Thion et al., 2018; Wolf et al., 2017). Communication between microglia and other cell types is mediated by motile microglial processes, through which microglia perform dynamic surveillance of their environment (Davalos et al., 2005; Haynes et al., 2006; Nimmerjahn et al., 2005). In addition to the well-documented microglia–neuron interactions, microglial processes are recruited to blood vessels and cells of the neurovascular unit (NVU), although the function of these interactions has remained vaguely defined (Benyo et al., 2016; Dudvarski Stankovic et al., 2016; Zhao et al., 2018). Microglia–vascular interactions are present in the brain from early development into adulthood, through which microglia regulate blood–brain barrier (BBB) permeability, leukocyte extravasation, and angiogenesis (Dudvarski Stankovic et al., 2016; Jolivel et al., 2015; Lou et al., 2016). In fact, microglia and microglial processes are closely associated with developing blood vessels in the neuroepithelium or in the ventricular zone, while Pu.1^−/−^ mice or Csf1^op/op^ mice that lack microglia and macrophages display impaired angiogenesis in the retina (Arnold and Betsholtz, 2013; Dixon et al., 2021; Dudvarski Stankovic et al., 2016; Penna et al., 2021). In the adult brain, CD206-positive perivascular macrophages (PVMs) remain closely associated with blood vessels, while CD206-negative microglial cell bodies occupy the brain parenchyma isolated by the glia limitans (Goldmann et al., 2016; Kida et al., 1993; Ransohoff and Engelhardt, 2012). PVMs have recently been shown to play an important role in neurovascular dysfunction associated with hypertension, via promoting BBB permeability (Faraco et al., 2016). However, little is known about the possible contribution of microglia to cerebral blood flow (CBF) or perfusion deficits in the adult brain.

Microglia respond to vascular injury, as seen in experimental models of stroke, Alzheimer’s disease, or multiple sclerosis (Dudvarski Stankovic et al., 2016; Zhao et al., 2018), and microglial processes are recruited to sites of BBB leakage within minutes (Davalos et al., 2012; Jolivel et al., 2015; Lou et al., 2016). However, we could not detect major differences in the extent of BBB injury after experimental stroke in the absence of microglia (Szalay et al., 2016). Interestingly, changes in microglial process dynamics around capillaries are proportional to the level of CBF reduction during transient ischemia (Masuda et al., 2011), suggesting a possible role for microglia–vascular interactions beyond vascular injury.

We have recently identified specific sites on neuronal cell bodies through which microglia shape neuronal responses via purinergic mechanisms (Cserep et al., 2019). Because microglia interact with both neurons and blood vessels (Szalay et al., 2016) and neuronal activity–dependent changes in CBF are precisely controlled via multicellular interactions in the NVU (Iadecola, 2017), we argued that microglia are ideally positioned to sense and influence neurovascular responses under normal conditions or when the balance between oxygen/nutrient demand and blood supply is disturbed. Supporting this, inflammatory changes and altered microglial activity together with impaired CBF and neurovascular coupling often precede symptom onset in common neurological disorders (Iadecola, 2017; Kisler et al., 2017). Here, by using microglia depletion, transgenic mice, and pharmacological interventions, we identify microglia as important modulators of CBF during neurovascular coupling, hypercapnia-induced vasodilation, and adaptation to hypoperfusion in the cerebral cortex.

## Results

### Microglia form dynamic purinergic contacts with cells in the NVU that regulate CBF

**Figure 1.**
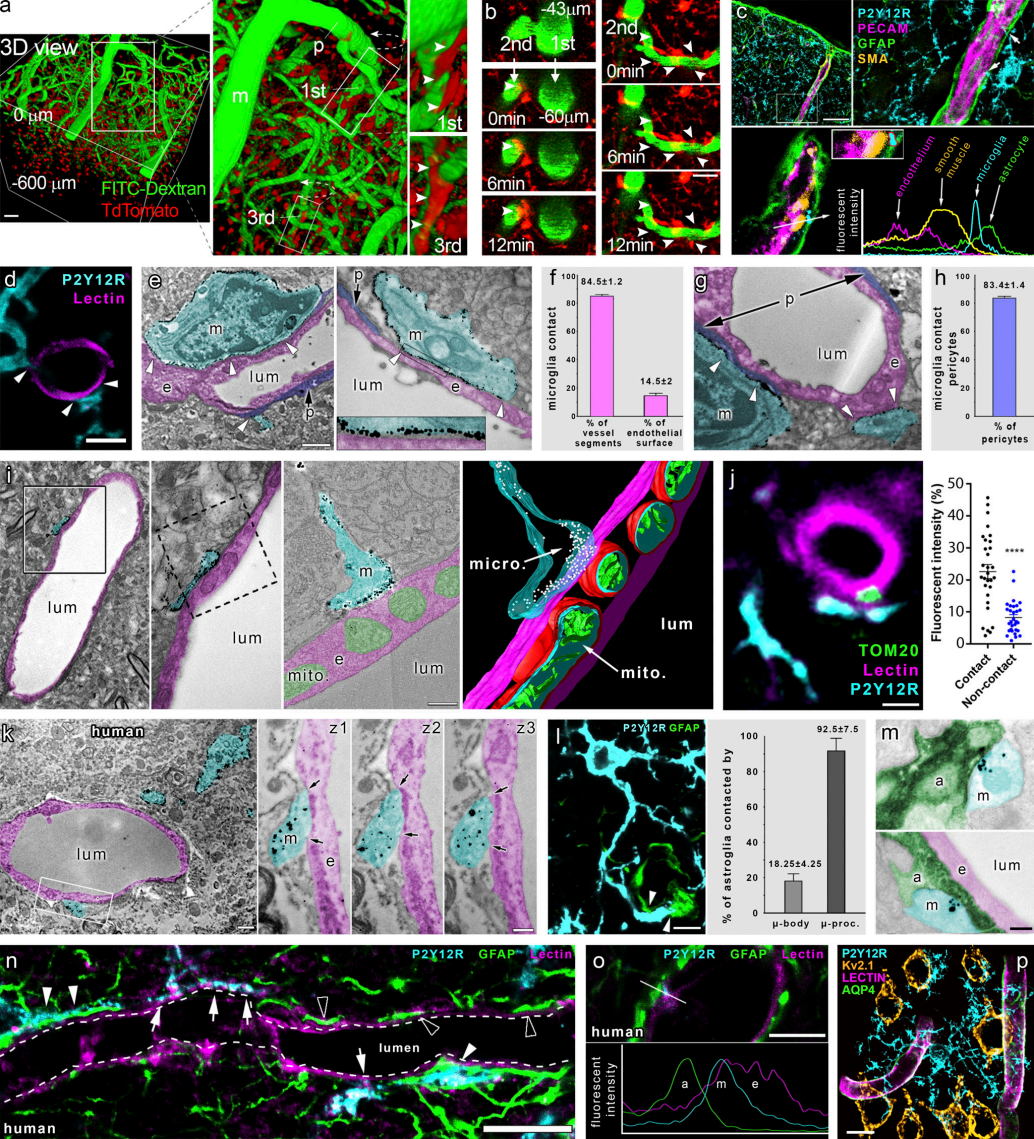
**Microglia form direct purinergic contact with cells in the NVU that regulate CBF. (a)** 3D reconstruction of in vivo two-photon Z-stacks down to 600 μm below the dura mater in the cerebral cortex of CX3CR1^tdTomato^ mice. Note contacting microglia (arrowheads) at meningeal (m), penetrating (p), and first- to third-order capillaries. Scale bar, 50 μm. **(b)** Microglial processes (arrowheads) dynamically contact different segments of the vascular tree (visualized by i.v. FITC-dextran). Scale bar, 20 µm. **(c)** Microglial processes are extended beyond the perivascular glial endfeet and form direct contact with smooth muscle cells (arrows) at the level of penetrating arteries. **(d)** CLSM images show microglia (P2Y12R, cyan) contacting endothelial cells (tomato lectin, magenta) in the cerebral cortex. **(e)** EM images show microglia (m, P2Y12R-immunogold labeling, cyan) directly contacting endothelial cells (e, magenta) and pericytes (p, purple). **(f)** Frequency of vessels receiving microglial contact, and microglial process coverage of endothelial cell surface. **(g)** EM images show microglia (m, P2Y12R-immunogold labeling, cyan) directly contacting pericytes (p, purple). **(h)** 83.4 ± 1.4% of pericytic cell bodies are contacted by microglial processes. **(i)** 3D reconstruction of electron tomogram shows clustering of anti-P2Y12R-immunogold on microglial processes (m) directly contacting the endothelium (e) of an arteriole/postcapillary venule. The left two panels are conventional EM images of the same area on the adjacent ultrathin section. The right panels show a tomographic virtual section and 3D reconstruction of the direct contact. **(j)** Unbiased anatomic analysis reveals enrichment of endothelial mitochondria (TOM20^+^, green), at sites of microglial contacts (P2Y12R^+^, cyan). ****, P < 0.0001, Mann–Whitney *U* test. See analysis details in Fig. S1 d. **(k)** EM images show microglia (m, P2Y12R-immunogold, cyan) directly contacting endothelial cells (e, magenta) in human neocortex. z1–z3 panels show the contact on three consecutive ultrathin sections; arrows mark the edges of direct membrane contact. **(l)** CLSM image shows microglia (P2Y12R, cyan) contacting the cell body of an astrocyte (GFAP labeling, green) and astrocytic endfeet (arrowheads). **(m)** EM images show direct contact between microglial (m, cyan) and astrocytic (a, green) processes. e, endothelial cell, magenta; lum, lumen. **(n)** CLSM images in human neocortex reveal P2Y12R^+^ microglial processes (cyan) contacting perivascular astrocytes (GFAP, green) on astrocyte endfeet (white arrowheads) and endothelial cells (tomato-lectin, magenta, white arrows), with astrocytic endfeet directly touching the endothelial monolayer (empty arrowheads). **(o)** CLSM image and fluorescent intensity plots show microglial process (m) contacting the endothelial layer (e) within the astrocytic layer (a). **(p)** To reveal 3D connections of individual microglial cells, a CLSM maximum-intensity plot was generated. Microglia (cyan) contact several microvessels (lectin, magenta; AQP4, green) and neurons (Kv2.1, ochre) simultaneously. For appropriate visualization of neuronal cell bodies, Kv2.1 is shown in yellow pseudocolor only with the maximal diameter planes included. Scale bars: (c) 50 µm; (d) 3 µm; (e) 2 µm; (i) 200 nm; (j) 2 µm; (k) left, 1 μm, and z3, 400 nm; (l) 10 µm; (m) 200 nm; (n) 10 µm; (o) 5 µm; (p) 10 µm.

**Figure S1.**
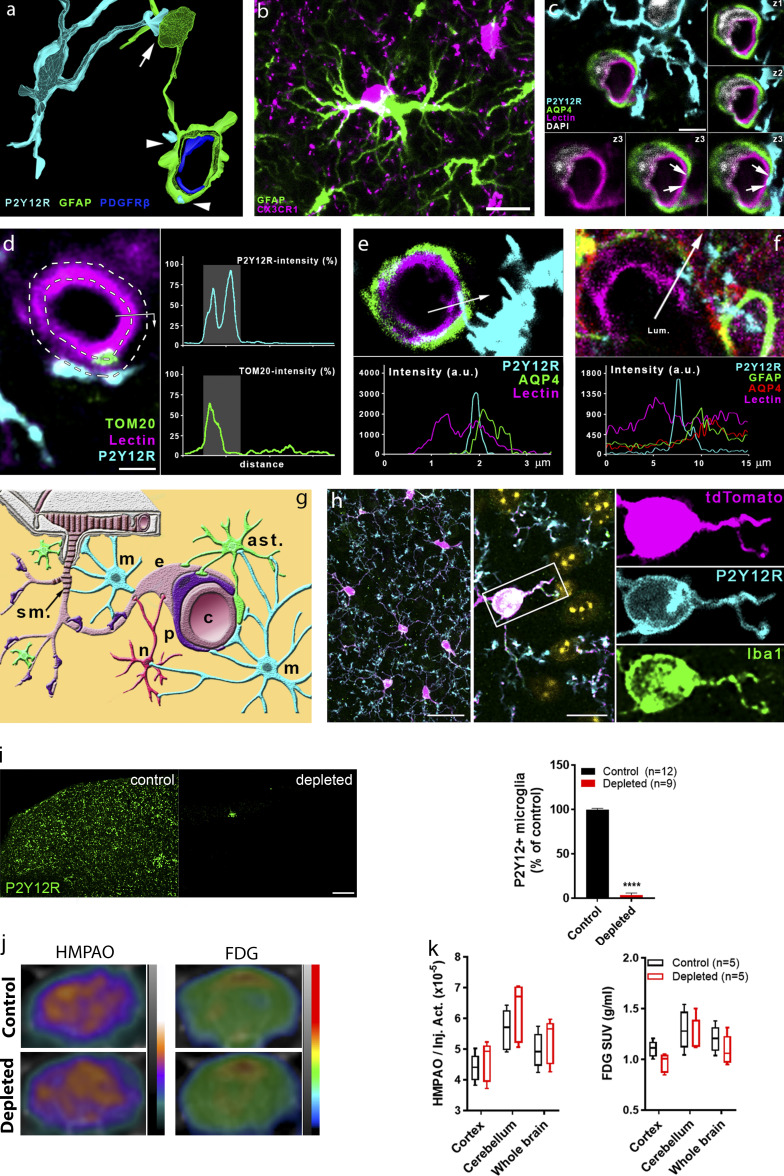
**Microglia form direct contacts with cells in the NVU, but microglia depletion does not disrupt cerebal perfusion or metabolism.** **(a)** 3D reconstruction of a high-resolution CLSM Z-stack shows microglia (P2Y12R, cyan) contacting both the cell body of an astrocyte (GFAP labeling, green, arrow) and astrocytic endfeet (arrowheads) ensheathing a capillary. Pericytes are visualized by anti-PDGFRb labeling (blue). **(b)** Microglia (CX3CR1, magenta) are able to form direct contact by their cell body with astrocytes (GFAP labeling, green) in the cerebral cortex. Scale bar, 10 μm. **(c)** CLSM image shows P2Y12R-positive microglial process (cyan) contacting perivascular AQP4-positive astrocyte endfeet (green) and also extends to the endothelial layer (lectin, magenta) where astrocytic coverage is not present (arrows). z1–z3 panels show the contact area on three consecutive confocal sections. Scale bar, 3 μm. **(d)** The process of semiautomated unbiased analysis of fluorescent intensity area for the graph presented in Fig. 1 j is depicted. White dashed lines represent the outer and the inner profiles, based on the outline of the endothelial cell. P2Y12R intensity was measured along the outer profile and TOM20 intensity along the inner profile, starting from the arrow. The intensity values are plotted (right) along the perimeter of the vessel. Contact site (marked by the gray column in the plots) was defined automatically. Scale bar, 2 µm. **(e)** CLSM image and fluorescent intensity plots show microglial process extending beyond perivascular astrocytic endfeet to interact with the endothelium. The fluorescent intensity profile plot (measured along the 3.5-µm long white arrow) clearly shows the presence of the microglial process under the astrocytic endfeet. **(f)** CLSM image and fluorescent intensity plots show microglial processes interacting with GFAP- and AQP4-positive astrocytes in the human brain. The fluorescent intensity profile plot (measured along the 15-μm-long white arrow) clearly shows the presence of the microglial process between the endothelium and the endfeet of perivascular astrocytes. **(g)** Schematic summary of contacts formed between microglia (m; cyan) and different cell types of the NVU. Neurons (n; red), astrocytes (ast.; green), pericytes (p; purple), endothelial cells (e; pale crimson), and vascular smooth muscle cells (s.m.; dark crimson) are shown. **(h**) Characterisation of CX3CR1^tdTomato^ mice. Parenchymal tdTomato-positive cells coexpress Iba1 and P2Y12R in the cerebral cortex. Cell nuclei stained with DAPI appear in yellow pseudocolor in the merged middle image. Scale bars, 25 µm (left), 10 µm (middle). **(i)** Feeding C57BL/6J mice with a diet containing PLX5622 results in an almost complete (97%) elimination of resident microglia as evidenced by the numbers of P2Y12R-positive cells in the cerebral cortex. Scale bar, 100 μm. *n* = 12 control and *n* = 9 depleted mice per group; ****, P < 0.0001 control versus depleted, unpaired *t* test with Welch’s correction. **(j and k)** HMPAO-SPECT and FDG-PET images of control and microglia-depleted mice. Proportion of measured and injected HMPAO activity (Inj. Act.) and standard uptake values (SUVs) of FDG are shown. Atlas-based ROI analysis (j) shows no significant differences between the normalized regional uptake values (k) of the two groups. *n* = 5 and 5 mice. Data are expressed as mean ± SEM.

We first investigated the formation and dynamics of microglia–vascular interactions using in vivo two-photon imaging. Intravenous FITC-dextran administration in CX3CR1^tdTomato^ microglia reporter mice allowed three-dimensional (3D) reconstruction of penetrating arterioles in the cerebral cortex down to 600 μm below the dura mater (Fig. 1 a). In vivo imaging revealed microglia ensheathing arterial bifurcations at the level of first-, second-, and third-order vessels and identified contacting microglial processes at all levels of the vascular tree (Fig. 1, a and b; and Video 1). The average lifetime of contacts ranged from 5 to 15 min, and microglial processes frequently recontacted the same sites at both arterioles and microvessels, suggesting that specific sites for microglia–vascular interactions may exist in the brain. Next, we studied the formation of physical contact between microglia and other cells in the NVU, using the microglial marker P2Y12 receptor (P2Y12R), which is not expressed by any other cells in the brain (Butovsky et al., 2014). Surprisingly, we found that processes of parenchymal microglial cells were extended beyond glial fibrillary acidic protein (GFAP)–positive perivascular glial endfeet at the level of penetrating arterioles, directly contacting smooth muscle actin (SMA)–positive smooth muscle cells (Fig. 1 c) and endothelial cells in both large vessels and microvessels as evidenced by both confocal laser scanning microscopy (CLSM) and immuno–electron microscopy (immuno-EM; Fig. 1, d and e). 3D analysis of Z-stacks recorded by CLSM revealed that 85% of blood vessel segments are contacted by microglial processes and 15% of the endothelial cell surface is covered by microglial processes (Fig. 1 f). The vast majority of pericytes (83%) also received direct microglial contact (Fig. 1, g and h). ATP (and ADP) is a major chemotactic factor for microglia via P2Y12R (Haynes et al., 2006), and purinergic signaling in endothelial cells and pericytes markedly influences CBF (Lohman et al., 2012). We have recently shown that clustering of microglial P2Y12R occurs at sites of somatic ATP release in neurons, through which microglia sense and influence neuronal activity and mitochondrial function (Cserep et al., 2019). To study whether ATP released at the perivascular compartment could also act as a chemotactic signal for microglial processes, we turned to 3D electron tomography. Importantly, we found contacting P2Y12R-positive microglial processes in close apposition with endothelial mitochondria, while immunogold particles were enriched at the interface (Fig. 1 i). Unbiased immunofluorescent analysis revealed 214% higher TOM20 immunofluorescence (a mitochondrial marker) in endothelial cells at microglial contact sites (Figs. 1 j and S1 d). Importantly, immuno-EM also confirmed the direct contact between P2Y12R-positive microglial processes and endothelial cells in the human brain (Fig. 1 k). The perivascular endfeet of GFAP expressing astrocytes contribute to CBF regulation (Howarth et al., 2017). We found that 93% of astrocytes were contacted by P2Y12R-positive microglial processes, while microglial cell bodies were found directly attached to 18% of astrocytes (Fig. 1 l; and Fig. S1, a and b). To visualize perivascular astrocyte endfeet, aquaporin-4 (AQP4) immunostaining was used, showing that microglial processes directly contact endothelial cells at sites void of the AQP4 signal (Fig. S1 c) or by extending through the astrocytic endfeet layer (Fig. S1 e). Combined immunogold-immunoperoxidase labeling and EM confirmed the direct contact between microglial processes and parenchymal astrocytes or perivascular astrocytic endfeet (Fig. 1 m). Similar observations were made in the human cerebral cortex from both aged and middle-aged patients who died in nonneurological conditions: P2Y12R-positive microglial processes established contact with both perivascular astrocyte endfeet and the endothelial monolayer of small arterioles and capillaries (Fig. 1, n and o; and Fig. S1 f). Furthermore, we found that individual microglial cells contact multiple microvessels and nearby neurons simultaneously in the brain (Fig. 1 p). Thus, not only do microglial processes directly contact cells in the NVU along the vascular tree (Fig. S1 g), which are known to shape CBF (Attwell et al., 2010; Hall et al., 2014; Iadecola, 2017; Kisler et al., 2017; Takano et al., 2006), but simultaneous contacts with neurons and vascular structures may provide an ideal platform for microglia to influence neurovascular responses.

**Video 1.** **Cerebral blood vessels were visualized by i.v. FITC-Dextran administration in CX3CR1**^**tdTomato**^ **mice, and microglial process dynamics was investigated by in vivo two-photon microscopy along the vascular tree.** Refer to Fig. 1, a and b for further details. Fig. 1 b depicts blood vessels identical to those shown in the video file, with arrowheads indicating the contact surfaces between microglial processes and first- or second-order capillaries.

### Microglia contribute to neurovascular coupling via P2Y12R-mediated actions

**Figure 2.**
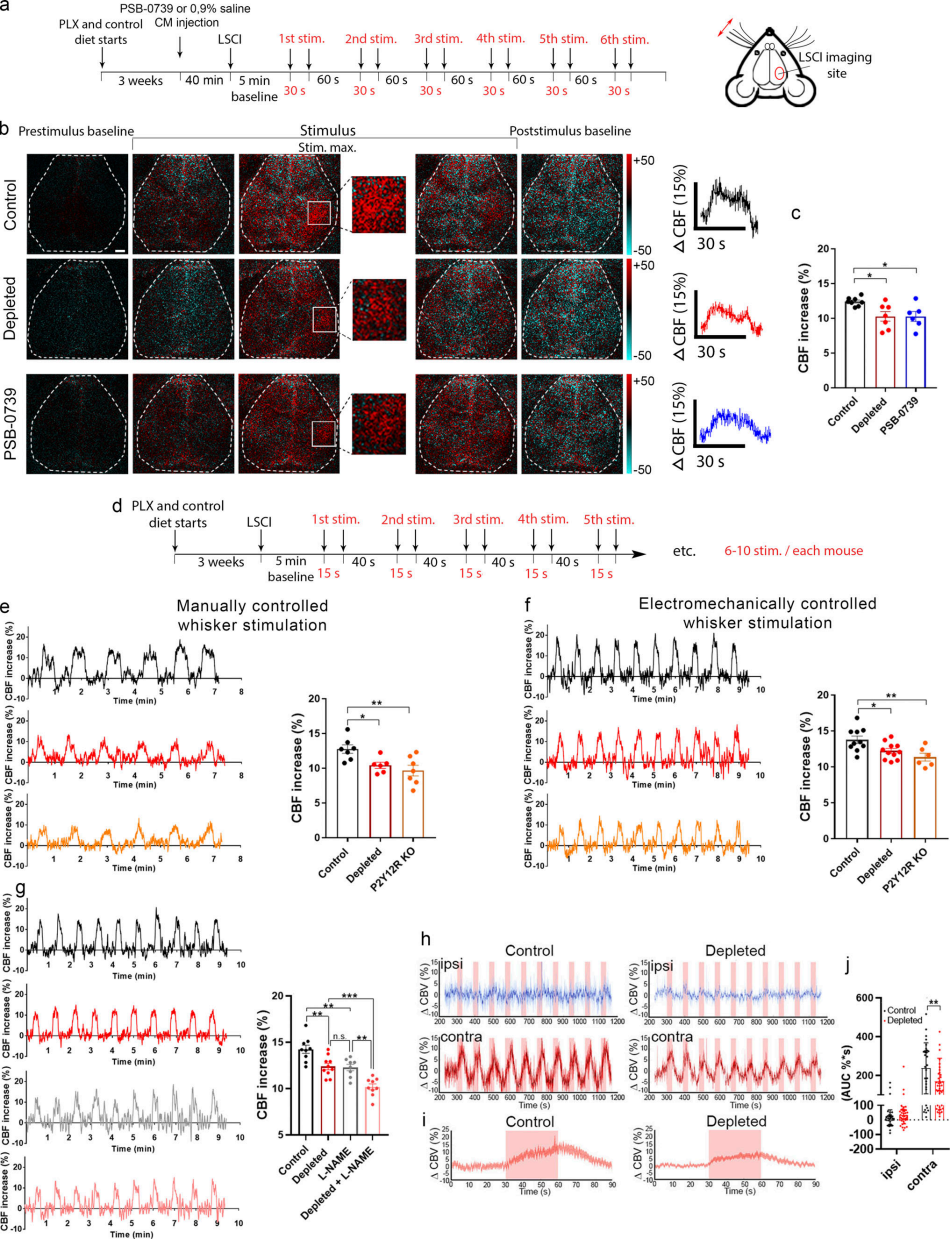
**Microglia contribute to neurovascular coupling in a P2Y12R-mediated manner. (a)** Schematic showing the outline of the experiment. **(b)** Difference images show CBF changes in the right barrel cortex relative to baseline in response to contralateral whisker stimulation before, during, and after stimulus (stim.; white rectangle indicates the barrel field). Time course of stimulus-evoked CBF responses is shown in the right of b. Scale bar, 1 mm. **(c)** Absence of microglia or acute blockade of P2Y12R reduces the maximum of evoked CBF responses compared with controls. *n* = 7 control, *n* = 7 depleted, and *n* = 6 PSB-0739 injected mice; *, P < 0.05, one-way ANOVA followed by Dunnett’s multiple comparison test (control versus depleted, P = 0.0191; control versus PSB-0739, P = 0.0243). **(d)** Protocol of manually and electromechanically controlled whisker stimulation. **(e and f)** Representative CBF traces and quantification show impaired neurovascular coupling response in the absence of microglia and in P2Y12R KO mice. *n* = 7 control, *n* = 6 depleted, and *n* = 7 P2Y12R KO mice (e and f); *n* = 10 control, *n* = 11 depleted, and *n* = 6 P2Y12R KO mice; **, P = 0.0075 (e); **, P = 0.0058 (f), one-way ANOVA followed by Dunnett’s multiple comparison test (e: *, P = 0.0378, control versus depleted; **, P = 0.0052 control versus P2Y12R KO; f: *, P = 0.0311 control versus depleted; **, P = 0.0047 control versus P2Y12R KO). **(g)** Representative CBF traces and graph show changes in neurovascular coupling response in L-NAME–treated mice in both the presence and the absence of microglia. *n* = 9 control, *n* = 10 depleted, *n* = 8 L-NAME–treated, *n* = 9 L-NAME–treated depleted; P < 0.0001, one-way ANOVA followed by Tukey’s multiple comparison test (**, P = 0.005, control versus depleted; **, P = 0.0049, control versus L-NAME; **, P = 0.0026, L-NAME versus depleted + L-NAME; ***, P = 0.0008, depleted versus depleted + L-NAME). **(h**) fUS imaging reveals reduced CBV responses compared with controls in the ipsilateral (ipsi) and contralateral (contra) barrel cortex. Representative traces of 10 subsequent stimulations (30 s each) are shown for control and microglia-depleted mice. **(i)** Peak trace averages of the contralateral side in control and depleted mice, with 95% confidence intervals. **(j)** Averaged AUC distribution for each group, as shown in pink window in i. Data are presented as mean ± SEM; *n* = 30 and *n* = 40 stimulations from three control and four depleted mice, respectively (j); **, P = 0.0093, two-way ANOVA followed by Sidak’s multiple comparisons test. Data are presented as mean ± SEM. LSCI data have been pooled from two to three independent experiments.

In our previous studies, we could not detect major alterations in the number or morphology of endothelial cells, astrocytes, or pericytes after elimination of microglia by CSF1R blockade (Szalay et al., 2016). To investigate whether prolonged absence of microglia could compromise overall vascular architecture or metabolism, we performed HMPAO-SPECT and FDG-PET measurements (Apostolova et al., 2012; Tai and Piccini, 2004) after microglia depletion by PLX5622 (Elmore et al., 2014). No significant changes in HMPAO or FDG uptake were observed in any brain areas after microglia depletion (Fig. S1, i–k). To investigate the role of microglia in CBF responses to physiological stimuli, we turned to the whisker-stimulation model, which is widely used to study the mechanisms of neurovascular coupling in mice (Tarantini et al., 2018) and performed laser speckle contrast imaging (LSCI), optimized to assess changes in the microcirculation through the intact skull bone in real time (Dunn, 2012). Whiskers on the left side were stimulated under mild ketamine-medetomidine sedation, allowing stable and reproducible CBF responses to be observed (Fig. 2, a and b). Surprisingly, the absence of microglia resulted in impaired functional hyperemia, as evidenced by a significant 15.3% reduction in CBF response in the right barrel cortex compared with that seen in control mice after a series of stimulation (six series of stimulations for 30 s each; Fig. 2, b and c; and Video 2). In addition, a 17% smaller CBF response to whisker stimulation was seen after acute microglial P2Y12R blockade by a specific P2Y12R inhibitor, PSB0739, injected into the cisterna magna 40 min before LSCI measurements (Cserep et al., 2019). Because only microglia express P2Y12R in the brain (Cserep et al., 2019; Fekete et al., 2018), this way we could also validate the specificity of microglial actions on CBF responses.

**Video 2.** **Representative LSCI videos showing the whisker stimulation–evoked reduced neurovascular coupling response in microglia-depleted mice compared with controls.** Difference images are shown, which display the stimulus-evoked CBF increase over baseline. White rectangles indicate the area of the barrel cortex.

To extend these observations with genetic P2Y12R blockade, another series of measurements were performed, using manual whisker stimulation followed by a set of electromechanically controlled stimulations. We found reduced CBF responses to whisker stimulation in both microglia-depleted and P2Y12R knockout (KO) mice, irrespective of the type of stimulation used (Fig. 2, d–f). Given the pivotal roles of NO in vasodilation, and specifically in neurovascular coupling (Attwell et al., 2010; Iadecola, 2017), we investigated the relationship between microglia depletion and nitric oxide synthase (NOS) blockade by L-NAME. Interestingly, we found that both L-NAME and microglia depletion significantly decreased the CBF response to whisker stimulation compared with control mice, while L-NAME combined with depletion had an additive effect (Fig. 2 g). These results suggest that in addition to microglial modulation of vasodilation in response to somatosensory stimulation, a microglia-independent NO-based component is also involved.

Finally, to confirm the role of microglia in neurovascular coupling with an alternative approach, we turned to functional ultrasound (fUS) imaging, which detects hemodynamic changes in the brain based on cerebral blood volume (CBV; Mace et al., 2011). We found that the absence of microglia resulted in significantly smaller (by 28%) CBV increases in the contralateral barrel cortex in response to whisker stimulation compared with that seen in control mice (Fig. 2, h–j). Thus, microglia-mediated and microglial P2Y12R–mediated actions are important to maintain normal blood flow responses to somatosensory stimulation in the cortical microcirculation, which is partially independent of NO.

### Changes in whisker stimulation–evoked neuronal responses do not explain altered CBF responses after microglia manipulation

**Figure 3.**
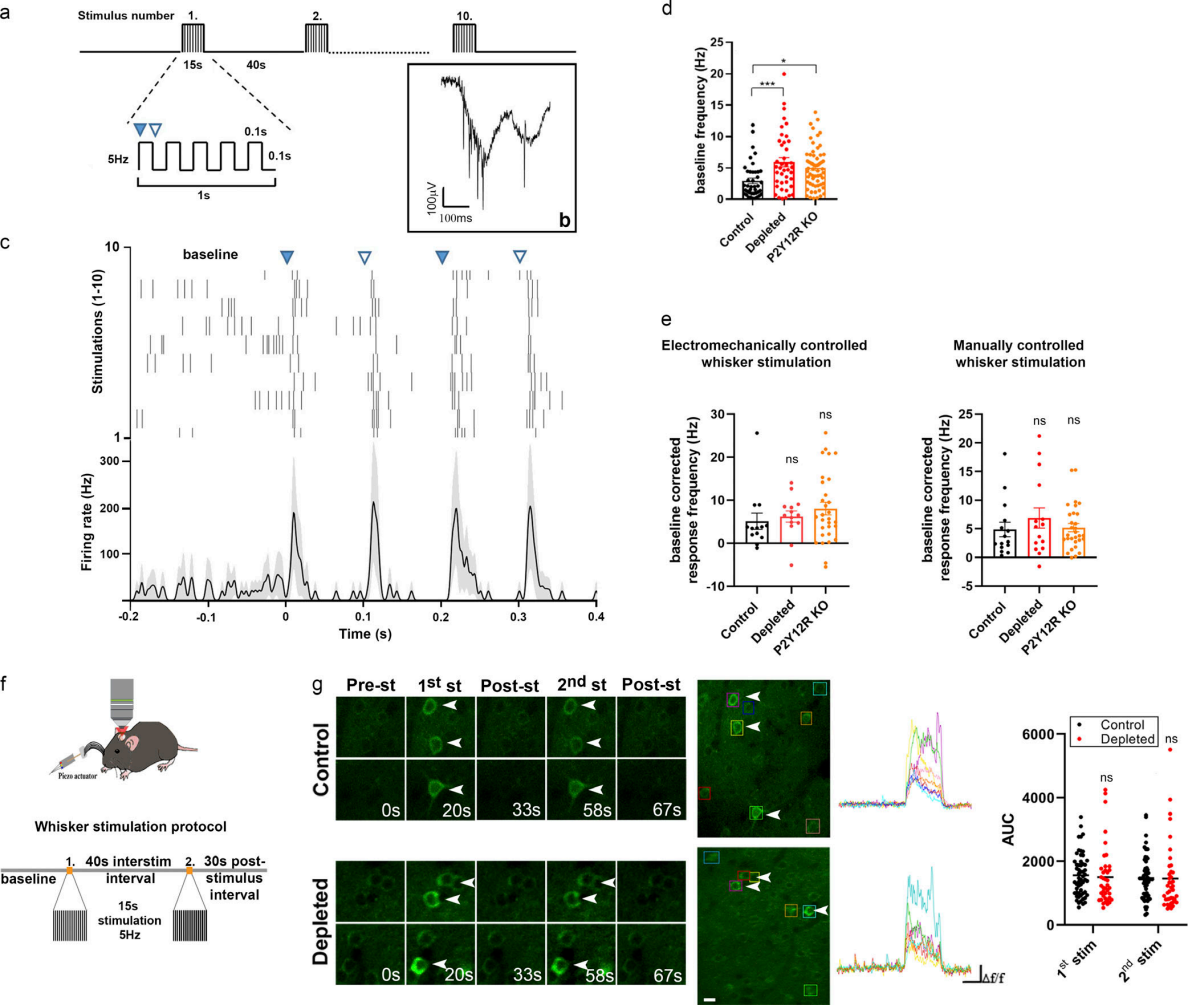
**Whisker stimulation–evoked neuronal responses in the barrel cortex do not explain altered CBF responses after microglia manipulation. (a)** Schematics of the whisker stimulation protocol. Whiskers were stimulated electromechanically with 5 Hz, causing alternating passive deflections of the vibrissae in the anterior and posterior directions (filled and empty arrowheads, respectively) for 15 s, followed by a 40-s pause, repeated 10 times. **(b)** Raw tetrode data showing extracellular spikes recorded from the barrel cortex. **(c)** Example of a single neuron activated by passive whisker deflections. Top, raster plot aligned to whisker stimulation onset (black ticks, individual action potentials). Bottom, peristimulus time histogram showing mean firing responses of the same neuron (shading, SEM). **(d)** Baseline firing rates were significantly higher in depleted and P2Y12R KO mice compared with controls. *n* = 4 control, *n* = 3 depleted, and *n* = 5 P2Y12R KO mice; P = 0.001, one-way ANOVA with Dunnett’s multiple comparisons test (***, P < 0.006, control versus depleted; *, P = 0.0109, control versus P2Y12R KO). **(e)** Stimulus-induced firing rate changes were comparable across controls and microglia-depleted mice using either electromechanically or manually controlled whisker stimulation. Data are presented as baseline corrected response frequency (for corresponding baseline frequencies, mean ± SEM); *n* = 4 control, *n* = 3 depleted, and *n* = 5 P2Y12R KO mice; P = 0.2087 and P = 0.6391, Kruskal–Wallis test with Dunn’s multiple comparisons test. **(f)** Schematic outlines of the whisker stimulation protocol used for in vivo two-photon [Ca^2+^]_i_ imaging in the barrel cortex of Thy1-GCaMP6s mice. interstim, interstimulation. **(g)** Representative images show stimulus-evoked neuronal [Ca^2+^]_i_ responses with individual traces of neurons labeled with rectangles during baseline imaging and 15-s stimulation and after stimulation. AUC values of neuronal GCaMP6s signal changes in response to the first and second electromechanically controlled whisker stimulation in control and microglia-depleted mice. *n* = 4 mice per group, *n* = 56 neurons from control and *n* = 40 neurons from depleted mice from two trials; P = 0.765, two-way ANOVA with Sidak’s multiple comparisons test. Scale bar, 20 µm. st and stim, stimulation. Data are presented as mean ± SEM.

To test whether substantial shifts in neuronal responses to whisker stimulation could explain the effect of microglia manipulation on functional hyperemia, we repeated these experiments while recording neuronal activity from the contralateral barrel cortex, using either chronically implanted tetrode electrodes or in vivo two-photon calcium imaging. We isolated *n* = 42, *n* = 41, and *n* = 61 putative single units from two electrophysiological recordings each of control, microglia-depleted, and P2Y12R KO mice, respectively (*n* = 5). This allowed us to test baseline firing rates in the stimulus-free periods as well as stimulus-induced firing responses of individual neurons. We found significantly increased baseline firing rates of barrel cortex neurons in both microglia-depleted and P2Y12R KO mice compared with controls (Fig. 3, a and d). However, using either electromechanically controlled automated whisker stimulation or manual stimulation, we did not detect differences in the extent of stimulus-evoked neuronal responses (Fig. 3 e).

Then, we turned to in vivo two-photon measurements, using electromechanical whisker stimulation, which was repeated two times with 40-s intervals (Fig. 3 f). Only neurons in the contralateral barrel cortex that specifically responded to both stimuli were selected for analysis. We found that somatosensory stimulus–induced increases in the neuronal GCaMP6s signal in Thy1-GCaMP6s mice did not reveal significant differences between control and microglia-depleted mice (Fig. 3 g and Video 3). Thus, while the absence (PLX5622 depleted) or dysfunction (P2Y12R KO) of microglia may shift baseline neuronal activity as expected (Badimon et al., 2020; Cserep et al., 2019), stimulus-evoked neuronal responses do not explain the marked differences in CBF changes observed after microglia manipulation.

**Video 3.** **Representative resonant in vivo two-photon imaging videos showing individual neuronal [Ca**^**2+**^**]**_**i**_ **responses to electromechanically controlled whisker stimulation in control and microglia-depleted Thy1-GCaMP6s mice.** Red dot indicates the onset of the individual stimuli (15 s long) repeated twice with 40-s intervals.

### Real-time chemogenetic modulation of microglial activity results in impaired functional hyperemia

**Figure 4.**
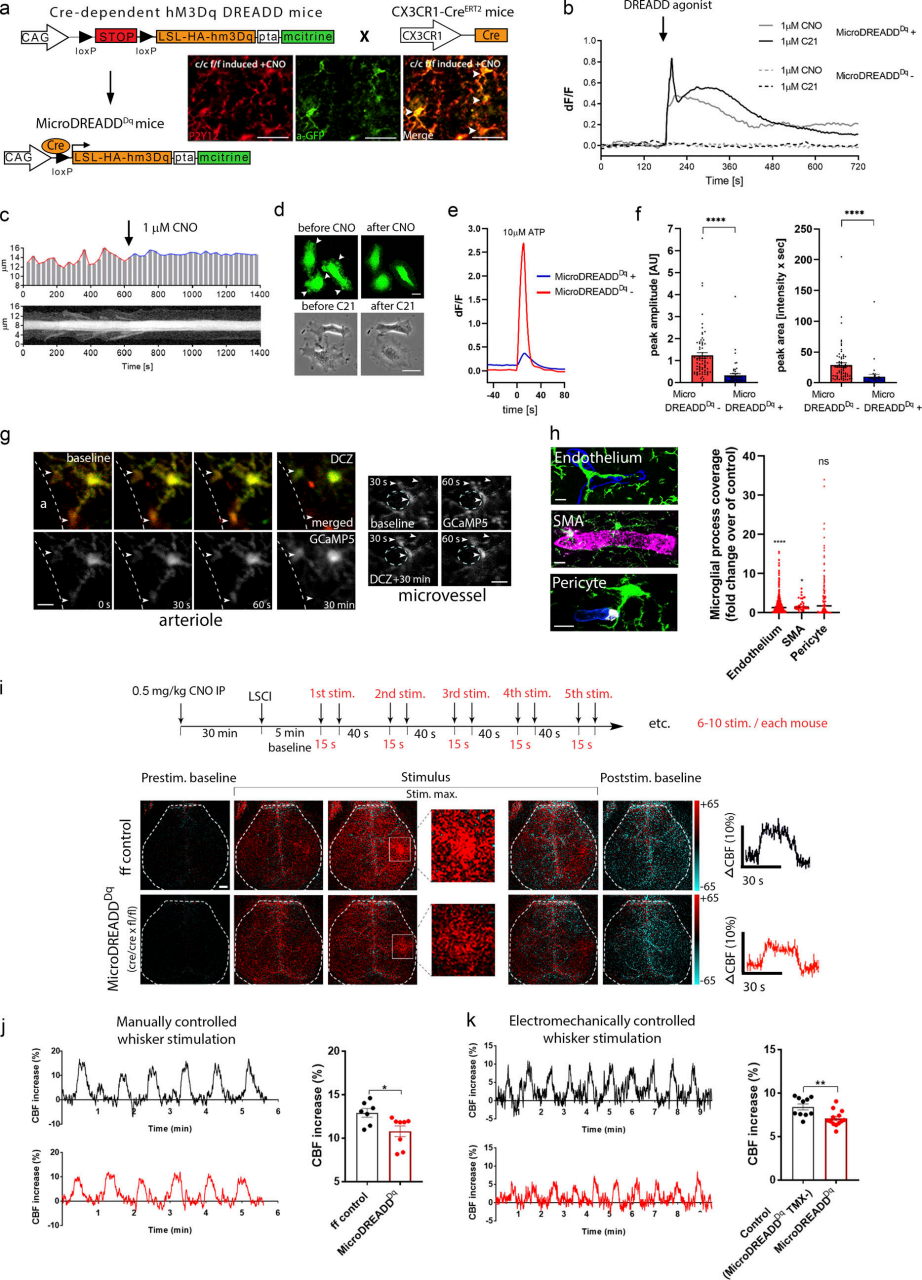
**Chemogenetic modulation of microglial activity leads to decreased process motility and impaired neurovascular coupling response to whisker stimulation. (a)** Generation of a novel chemogenetic mouse model. TMX-induced recombination was confirmed by anti-P2Y12R and anti-GFP (mCitrine) double staining (white arrowheads), allowing chemogenetic activation of microglia by CNO. Scale bar, 50 μm. **(b)** Representative ∆*F*/*F* calcium traces of MicroDREADD^Dq^ microglia cells responding to DREADD agonists CNO or C21. **(c and d)** The kymogram (c) and fluorescent/phase contrast images (d) taken from time-lapse sequences show that cell membrane ruffling is ceased upon treatment with DREADD agonists, and the cells acquire a flattened morphology. Scale bars, 5 μm, upper panel; 10 μm, lower panel. See also Video 4 and Video 5. **(e and f)** Analyses of calcium curves reveal an attenuated responsiveness to ATP in MicroDREADD^Dq+^ cells previously responding to C21. See details in Fig. S2. *n* = 64 for MicroDREADD^Dq+^, *n* = 73 for MicroDREADD^Dq−^; ****, P < 0.0001, Mann–Whitney *U* test (f). **(g)** Microglial processes interacting with blood vessels show dynamic [Ca^2+^]_i_ fluctuations (arrowheads) in the cerebral cortex of MicroDREADD^Dq^ × CGaMP5g–tdTomato mice in vivo. Microglial responses have been investigated before (baseline) and 30 min after administration of the DREADD agonist deschloroclozapine (DCZ) around arterioles (a, lumen of the arteriole is shown) and microvessels (*n* = 4 mice). Scale bar, 10 µm. **(h)** 1 h after chemogenetic activation, microglial process coverage (Iba1, green) of endothelial cells (lectin, blue), smooth muscle cells (SMA, magenta), and pericytes (PDGFRb, white) was assessed on perfusion fixed brain sections. Scale bar, 10 µm. *n* = 263 blood vessels, *n* = 66 SMA-positive vessels, and *n* = 291 pericytes were measured from *n* = 3 mice; ****, P = 0.0001 endothelium versus control and *, P = 0.026 SMA versus control, Mann–Whitney *U* test. **(i)** 6 wk after TMX, CBF was measured by LSCI during whisker stimulations in MicroDREADD^Dq^ and control mice 30 min after a single i.p. (IP) CNO administration. Representative difference images show CBF changes relative to baseline in control and MicroDREADD^Dq^ mice (white rectangle indicates the area of barrel cortex). Representative stimulus-evoked response curves are shown in the right of i. Scale bar, 1 mm. **(j and k)** Representative CBF curves of manually and electromechanically controlled whisker stimulation measured by LSCI. The maximum of evoked responses show a marked reduction in MicroDREADD^Dq^ mice compared with controls. *n* = 7 control and *n* = 8 MicroDREADD^Dq^ mice; *, P = 0.0401, Mann–Whitney *U* test (j); *n* = 10 control and *n* = 13 MicroDREADD^Dq^ mice; **, P = 0.0045, unpaired *t* test (k). Data are presented as mean ± SEM. LSCI data have been pooled from two to three independent experiments.

**Figure S2.**
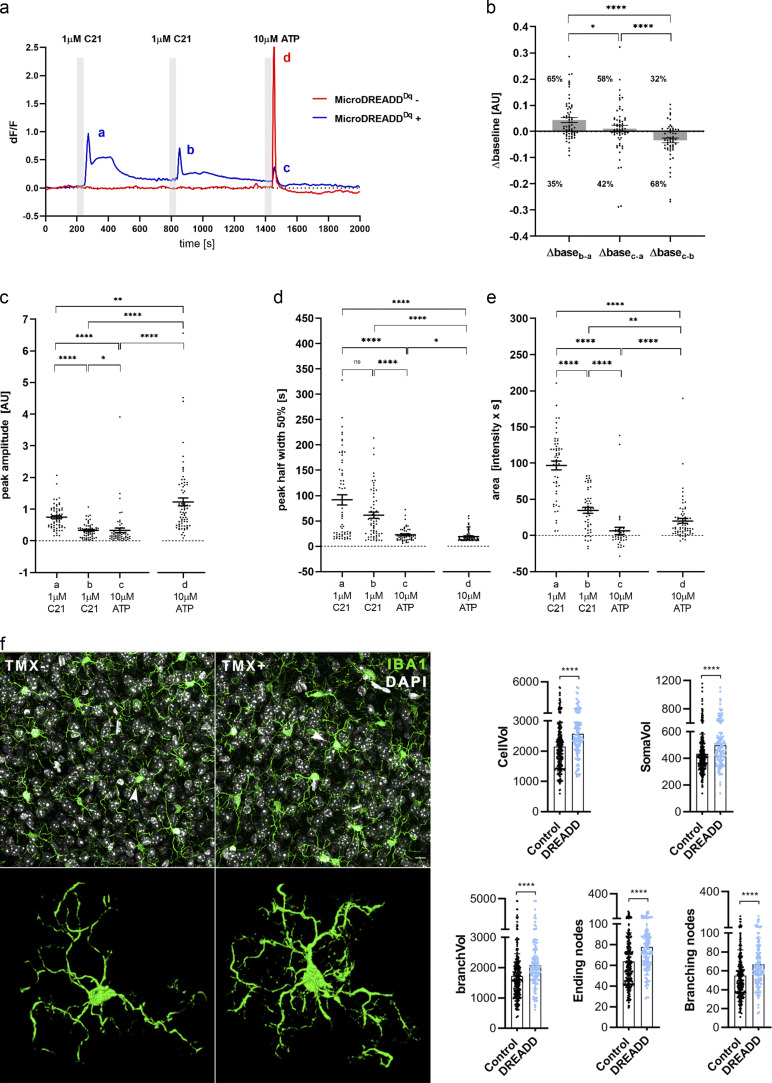
**Microglial cells expressing DREADD (hM3Dq) respond to DREADD agonist C21 with a biphasic [Ca^2+^]_i_** **response and reduced ATP responsiveness. (a)** Representative calcium signals of cultured MicroDREADD^Dq+^ and MicroDREADD^Dq−^ microglia cells repeatedly exposed to 1 µM C21 and 10 µM ATP for 1 min. **(b)** Differences of average baseline values determined within 50 s before the onset of the responses. Mann-Whitney *U* test; ^*^, P < 0.05; ****, P < 0.0001. **(c–e)** Peak amplitudes (c), half-width values (d), and peak areas (e) of *a*, *b*, *c* and *d* peaks denoted in the line graph (a). *n* = 64 for MicroDREADD^Dq+^, *n* = 73 for MicroDREADD^Dq−^; Mann–Whitney *U* test; ****, P < 0.0001; **, P < 0.01; *, P < 0.05 (c–e). **(f)** Automated analysis shows marked morphological changes in MicroDREADD^Dq+^ microglia in TMX-treated mice 1 h after i.p. CNO administration, compared with littermates in which hM3Dq DREADD expression was not induced with TMX (control). *n* = 275 control (MicroDREADD^Dq−^) and *n* = 122 DREADD (MicroDREADD^Dq+^) microglia from *n* = 3 mice from each group were analysed. Mann–Whitney *U* test; ****, P < 0.0001 for cell volume (CellVol), soma volume (SomaVol), branch volume (branchVol), ending nodes, and branching nodes. Scale bar, 20 μm. Data are expressed as mean ± SEM.

To explore the effect of real-time chemogenetic microglia manipulation on CBF changes, we generated a novel mouse line by crossing Cre-dependent hM3Dq DREADD mice with CX3CR1-CreERT2 mice (Ginhoux et al., 2010), named MicroDREADD^Dq^ mice. Tamoxifen (TMX) administration resulted in specific recombination in 95.3% of microglia (Fig. 4 a). Recombination based on the weak mCitrine signal is still likely to be underestimated, because using the identical CX3CR1-CreERT2 driver line, virtually 100% of Iba1/P2Y12R double-positive microglia were found to express tdTomato in the cerebral cortex of CX3CR1^tdTomato^ reporter mice (see Materials and methods and Fig. S1 h). HM3Dq DREADD agonists 1 μM clozapine-*N*-oxide (CNO) or 1 μM Compound 21 (C21; Thompson et al., 2018) induced rapid increases in intracellular calcium levels in microglia derived from MicroDREADD^Dq^ mice, which was completely absent in TMX-untreated cells (Fig. 4 b; and Fig. S2, a–e). Single chemogenetic activation led to the blockade of microglial process motility within a few minutes, while microglia showed reduced calcium responses to repeated C21 stimulations (Fig. 4, c and d; Fig. S2, a–e; and Videos 4 and Video 5). Chemogenetic stimulation also resulted in altered microglial morphology and branch structure in vivo (Fig. S2 f). Importantly, C21 stimulation markedly impaired responses of MicroDREADD^Dq^ microglia to 10 μM ATP (Fig. 4, e and f), suggesting that chemogenetic priming disables the recruitment of microglial processes to ambient ATP released in the NVU during functional hyperemia (Pelligrino et al., 2011).

**Video 4.** **Fluorescent time-lapse recording of cultured MicroDREADD**^**Dq**^ **microglia cells responding to 1 µM C21 DREADD agonist.** The cells were loaded with Cal590 AM fluorogenic calcium sensitive dye (red) and the cell membrane was stained with the FluoVolt membrane labeling dye (green). The video was recorded at 0.25 frame/s in a 150 × 107-µm field of view. Scale bar, 10 µm. Note decreased process motility already after the first C21 treatment and markedly decreased calcium responses to repeated C21 stimulations.

**Video 5.** **Phase-contrast video of MicroDREADD**^**Dq+**^ **microglia cells responding to 1 µM C21 DREADD agonist.** The video was recorded at 2 frame/min for 60 min on a Zeiss Axiovert 200M microscope in a 150 × 107-µm field of view. Scale bar, 10 µm. Note decreased process dynamics after C21 treatment.

To investigate microglial calcium dynamics and the effect of chemogenetic activation in vivo, we crossed MicroDREADD^Dq^ mice with Cre-dependent CGaMP5g–tdTomato mice (i.e., both constructs could be induced by TMX using the same CX3CR1–CreERT2 driver line). Interestingly, we found that microglial processes interacting with arterioles and microvessels in the cerebral cortex showed dynamic calcium fluctuations, as assessed by in vivo two-photon imaging (Fig. 4 g). Chemogenetic activation in MicroDREADD^Dq^ × CGaMP5g–tdTomato mice resulted in an average of 18% increase in microglial somatic CGaMP5g signal within 15–30 min, leading to the temporary detachment and withdrawal of a population of perivascular microglial processes, which was most apparent around arterioles. As a likely compensatory response, 1 h after chemogenetic activation, microglial process coverage increased around endothelial cells (P = 0.001) and smooth muscle cells (P = 0.026), whereas a nonsignificant trend was observed in process coverage of pericytes (Fig. 4 h). Importantly, we found that chemogenetic modulation of microglia 30 min before LSCI measurements resulted in a similar degree of CBF reduction to both manual and electromechanical whisker stimulation (Fig. 4, i–k), as seen after microglia depletion (Fig. 2, e and f).

### Microglia modulate hypercapnia-induced vasodilation in a P2Y12R-dependent manner

**Figure 5.**
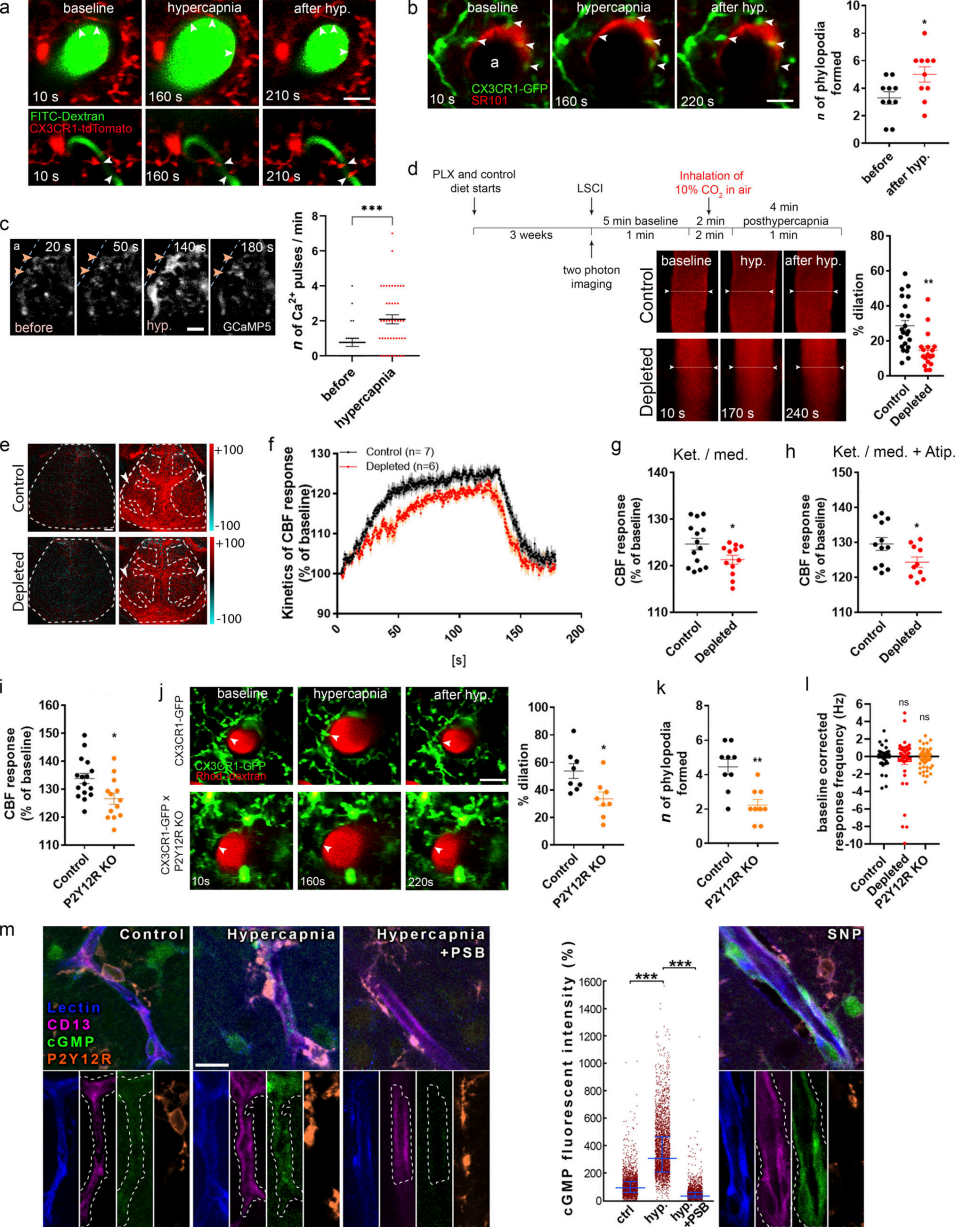
**Microglia contribute to hypercapnia-induced vasodilation. (a)** In vivo two-photon resonant (32-Hz) imaging was performed in the somatosensory cortex of CX3CR1^tdTomato^ mice during hypercapnia (by inhalation of 10% CO_2_ under normoxic conditions). The middle panel shows the maximal vasodilation provoked by hypercapnia. Scale bar, 20 μm. **(b)** Identical hypercapnic challenge and imaging protocol was performed in CX3CR1^GFP/+^ mice after intracortical injection of SR101 to visualize astrocytes. The number of phylopodia formed at the end of contacting microglial processes (arrowheads) increased in response to hypercapnia. *n* = 5 mice; *, P = 0.0316, Mann–Whitney *U* test. Scale bar, 20 µm. **(c)** Perivascular microglia respond rapidly to hypercapnia with [Ca^2+^]_i_ pulses in small (arrowheads) and large processes as assessed in CX3CR1^CGaMP5g–tdTomato^ mice. Individual processes were followed with in vivo two-photon resonant (31-Hz) imaging; see also Video 7. Scale bar, 10 µm. *n* = 4 mice; ***, P = 0.001, Mann–Whitney *U* test. **(d)** In vivo two-photon imaging reveals impaired vasodilation at the level of penetrating arteries in the absence of microglia. *n* = 22 and *n* = 18 vessels from eight control and six depleted mice; **, P = 0.0013, unpaired *t* test. The experimental protocol shown for hypercapnic (hyp.) challenge was identical for in vivo two-photon imaging (a–d and j–l) and LSCI (e–i). **(e)** Difference images show reduced CBF response in microglia-depleted mice to hypercapnic challenge (ROIs are labeled with arrowheads). Scale bar, 1 mm. **(f)** The average kinetics of hypercapnic responses show difference in depleted mice compared with controls. *n* = 14–12 ROIs from seven control and six depleted mice, two ROIs/mouse (f and g); ****, P < 0.0001, Mann–Whitney *U* test (f); *, P = 0.0472, unpaired *t* test (g). **(g and h)** Hypercapnia-evoked CBF response is markedly decreased in the absence of microglia under ketamine-medetomidine (Ket./med.; g) or Ket./med. (h) anesthesia after administration of atipamezole (Atip.). *n* = 12–10 ROIs from six control and five depleted mice, two ROIs/mouse; *, P = 0.0436, unpaired *t* test. **(i)** Hypercapnia-evoked CBF response is markedly decreased in P2Y12R KO mice as assessed by LSCI. *n* = 16 control, *n* = 13 P2Y12R KO; *, P = 0.0131, unpaired *t* test. **(j)** In vivo two-photon imaging reveals that elimination of P2Y12R impairs hypercapnia-induced vasodilation in double transgenic (CX3CR1^GFP/+^ × P2Y12R KO) mice compared with P2Y12R-competent CX3CR1^GFP/+^ mice. *n* = 8 and 8 vessels from *n* = 5 control and *n* = 5 P2Y12R KO mice; *, P = 0.0104, Mann–Whitney *U* test. Scale bar, 20 µm. **(k)** The number of phylopodia formed at the end of perivascular microglial processes in response to hypercapnia is significantly reduced in P2Y12R KO mice. *n* = 5 control and *n* = 5 P2Y12R KO mice; **, P = 0.003, Mann–Whitney *U* test. **(l)** During hypercapnic challenge, neuronal activity did not differ between control, microglia-depleted, and P2Y12R KO mice. *n* = 49 single units in control, *n* = 44 in depleted, and *n* = 61 in P2Y12R KO group; P = 0.4852, Kruskal–Wallis test with Dunn’s multiple comparison. **(m)** Single image planes for CLSM imaging show small blood vessel segments from the second to third layer of the neocortex in acute brain slices. Lectin (blue) outlines the vessels, CD13 labels contractile elements (pericytes and smooth muscle cells), microglial P2Y12R is orange, and cGMP signal can be seen in green. cGMP levels were measured within areas (outlined by white dashed line) masked based on CD13 staining. A low level of basal cGMP levels can be seen under control conditions, while hypercapnia induced a robust increase in vascular cGMP levels. Preincubation with the P2Y12R inhibitor PSB0739 abolished hypercapnia-induced cGMP elevation. As a control, application of the NO donor SNP also induced robust cGMP production. Scale bar is uniformly 15 µm. *n* = 3 mice; ***, P < 0.0001, Kruskal–Wallis test. Data are expressed as mean ± SEM (b–d and f–l) and median ± IQR (m). LSCI data have been pooled from two to three independent experiments.

**Figure S3.**
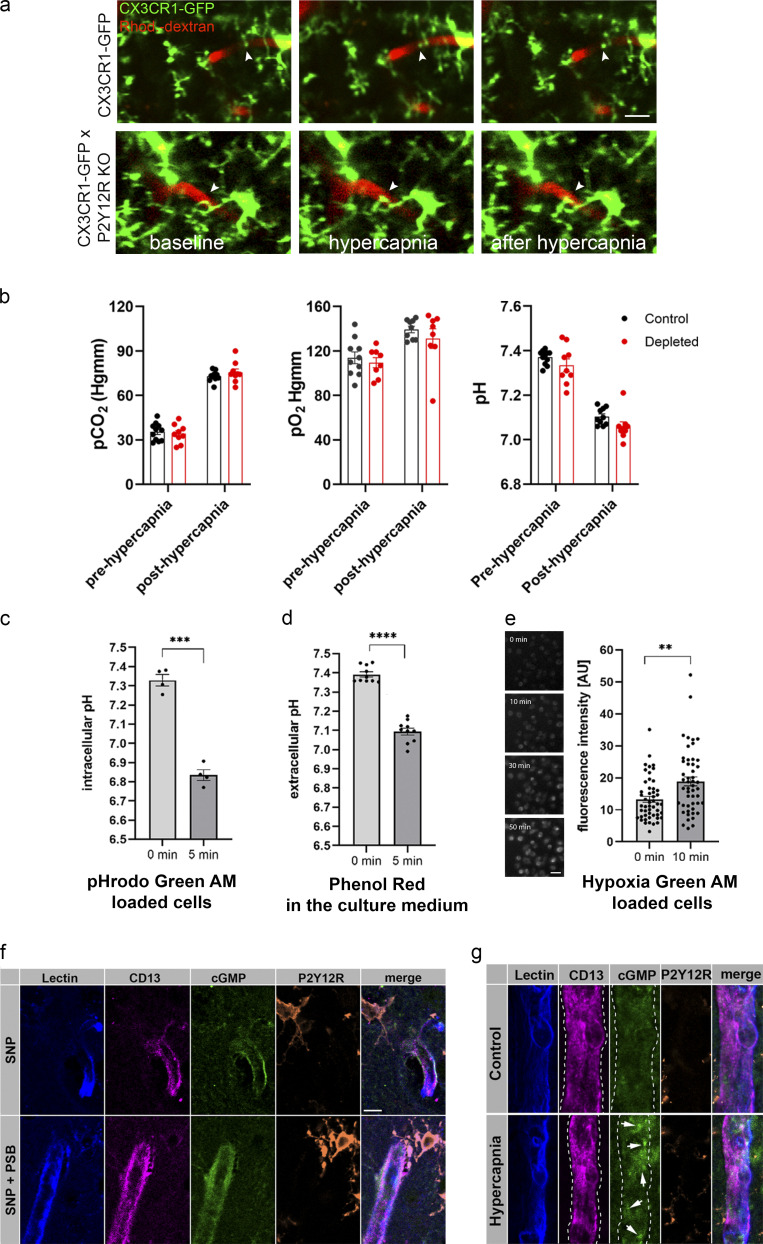
**Microglia modulation does not change blood gases but impacts on cGMP levels in the cerebral vasculature.** **(a)** In vivo two-photon imaging was performed with resonant scanning (32 Hz) in the somatosensory cortex of CX3CR1^GFP/+^ × P2Y12R KO and CX3CR1^GFP/+^ (P2Y12R-competent) mice following intravenous Rhodamine B-Dextran (Rhod.-dextran) administration to visualize blood vessels. After recording 60 s of baseline, vasodilation was induced by inhalation of 10% CO_2_ in air for 120 s (61–180 s) under normoxic conditions, followed by 60 s of posthypercapnia recording, using a protocol identical to that shown in Fig. 5 d. Scale bar, 10 µm. **(b)** Arterial pCO_2_, pO_2_, and pH measurements under ketamine-medetomidine anesthesia after the administration of atipamezole performed before and after hypercapnic challenge. Blood samples were taken from the femoral artery. No significant difference was observed between control and microglia-depleted mice. *n* = 10 control and *n* = 8 depleted mice, two-way ANOVA followed by Sidak’s multiple comparison test. **(c and d)** Both intracellular (c) and extracellular (d) pH markedly decreases within a few minutes after exposing cells to 15% CO_2_/85% air gas mixture, as a model of hypercapnia. Extracellular pH was determined by Phenol Red absorbance measurements, and intracellular pH was measured as changes in pHrodo Green AM dye fluorescence in glial cells. *n* = 4 parallels per group; ***, P = 0.0001, 0 min versus 5 min, paired *t* test (c); *n* = 10 parallels per group; ****, P < 0.0001, 0 min versus 5 min, paired *t* test (d). **(e)** Hypoxia Green AM loaded cells exhibit significant increase in fluorescent intensity within 10 min after placing microglia cultures to hypoxic environment (1% O_2_/5% CO_2_/94% N_2_). The reagent begins to fluoresce when oxygen levels drop below 5%. *n* = 50 parallels per group; **, P = 0.0019 0 min versus 10 min, Mann–Whitney *U* test. Scale bar, 30 µm. Data are shown as mean ± SEM (b–e). **(f)** Single image planes for CLSM imaging show small blood vessel segments from second and third layer of the neocortex in acute brain slices. Lectin (blue) outlines the vessels, CD13 labels contractile elements (pericytes and smooth muscle cells), microglial P2Y12R is orange, and cGMP signal can be seen in green (arrows). Note that PSB0739 treatment has no effect on SNP-induced cGMP. **(g)** CLSM imaging shows small blood vessel segments from the second and third layer of the neocortex in perfusion-fixed brain sections. Hypercapnia was induced in vivo and maintained in anesthetized mice until sacrifice. Lectin (blue) outlines the vessels, CD13 labels contractile elements (pericytes and smooth muscle cells), microglial P2Y12R is orange, and cGMP signal can be seen in green (arrows).

To further investigate the mechanisms through which microglia modulate CBF, we turned to hypercapnic challenge to induce vasodilation independently of direct neuronal stimulation. Hypercapnia is considered to induce primarily endothelium-driven vasodilation, including actions of astrocytes and other cells (Faraci et al., 2019; Hamilton et al., 2010; Howarth et al., 2017; Meng and Gelb, 2015; Yoon et al., 2012). In vivo two-photon imaging revealed a population of dynamic microglial processes that readily changed their morphology at both arterioles and microvessels in response to vasodilation induced by 2-min inhalation of 10% CO_2_ under normoxic conditions (Figs. 5 a; and S3 a). Around arterioles, SR101-labeled perivascular astrocyte endfeet were also dynamically contacted by microglia (Fig. 5 b), and the number of contacting phylopodia at the end of microglial processes increased in response to hypercapnia (Fig. 5 b). Confirming the rapid effect of hypercapnia on microglial process dynamics, we also found that perivascular microglia responded rapidly (within 1–2 min) to hypercapnia with calcium pulses as assessed in CX3CR1^CGaMP5g–tdTomato^ mice with in vivo two-photon imaging. Calcium pulses were apparent in both phylopodia and large processes (Fig. 5 c and Video 7). Importantly, in vivo two-photon imaging revealed significantly impaired hypercapnia-induced vasodilation in meningeal and penetrating arteries (Fig. 5 d), which paralleled smaller CBF responses in microglia-depleted mice as assessed by LSCI (Fig. 5, e–g). To exclude the potential effect of α2 adrenergic blockade via the cardiovascular system during ketamine-medetomidine anesthesia (Janssen et al., 2004), we repeated hypercapnic challenge after the administration of atipamezole, an α2 receptor antagonist (Pal et al., 2020). Hypercapnia-induced CBF response was similarly reduced in microglia-depleted mice compared with controls in the presence of atipamezole (Fig. 5 h). Importantly, baseline and hypercapnia-induced pCO_2_ and pO_2_ levels and pH in blood samples taken from the femoral artery were not altered by microglia depletion (Fig. S3 b). Similar to that seen after microglia depletion, decreased CBF response to hypercapnia was also apparent in P2Y12R KO mice compared with controls as measured by LSCI (Fig. 5 i). In vivo two-photon imaging also revealed 37% smaller hypercapnia-induced vasodilation in the absence of microglial P2Y12R (using CX3CR1^GFP/+^ × P2Y12R KO mice), compared with control (CX3CR1^GFP/+^) mice (Fig. 5 j and Video 6). Supporting the important role of microglial process interactions with the vasculature, formation of perivascular phylopodia was also significantly reduced after hypercapnia in P2Y12R KO mice (Fig. 5 k). In contrast, neuronal activity did not differ between control, microglia-depleted, and P2Y12R KO mice during hypercapnic challenge (Fig. 5 l).

**Video 6.** **Representative in vivo two-photon imaging videos recorded by the resonant scanner showing reduced hypercapnia-induced vasodilation in P2Y12R KO (CX3CR1**^**GFP/+**^ **× P2Y12R KO) mice compared with control (CX3CR1**^**GFP/+**^**) mice.** Blood vessels were visualized by administration of Rhodamine B-dextran. Hypercapnia was induced at 60 s and maintained until 180 s with a 60-s-long posthypercapnic period. Identical fields of view are shown in Fig. 5 j.

**Video 7.** **Representative in vivo two-photon resonant imaging video recorded in the cerebral cortex of CX3CR1**^**CGaMP5g–tdTomato**^ **mice during hypercapnic challenge (shown in ****Fig. 5 c****).** Perivascular microglial processes show dynamic [Ca^2+^]_i_ activity changes in response to hypercapnia. The arterial segment on the left is labeled with “a.”

Finally, we investigated the possible links between microglial P2Y12R and NO in hypercapnia-induced vasodilation. NO functions, including vasodilation, are mediated by cGMP, which is directly activated by NO (Toda et al., 2009). To achieve precise timing of hypercapnia, we prepared neocortical acute slices from mice and induced hypercapnia by bubbling 14.6% CO_2_ under normoxic conditions for 15 min before measuring cGMP immunoreactivity on rapidly fixed brain slices. Hypercapnia induced a robust increase of cGMP levels (to 311% of control values) in CD13-positive cells, a marker known to homogeneously label both smooth muscle ensheathing and thin-strand/mesh pericytes from large vessels to capillaries (Smyth et al., 2018). Importantly, increases in cGMP levels were markedly inhibited by the blockade of microglial P2Y12R with PSB0739 (Fig. 5 m). Confirming that indeed the NO-sGC-cGMP pathway caused the large increase of cGMP levels, the NO donor sodium nitroprusside (SNP) resulted in marked increases in identical anatomic structures, which was not affected by P2Y12R blockade (Fig. S3 f). Hypercapnia also increased cGMP in CD13-positive profiles in vivo (Fig. S3 g).

### Stimulus-specific release of purinergic metabolites by NVU cells parallels microglial modulation of brain pH and hypercapnia-induced adenosine production

**Figure 6.**
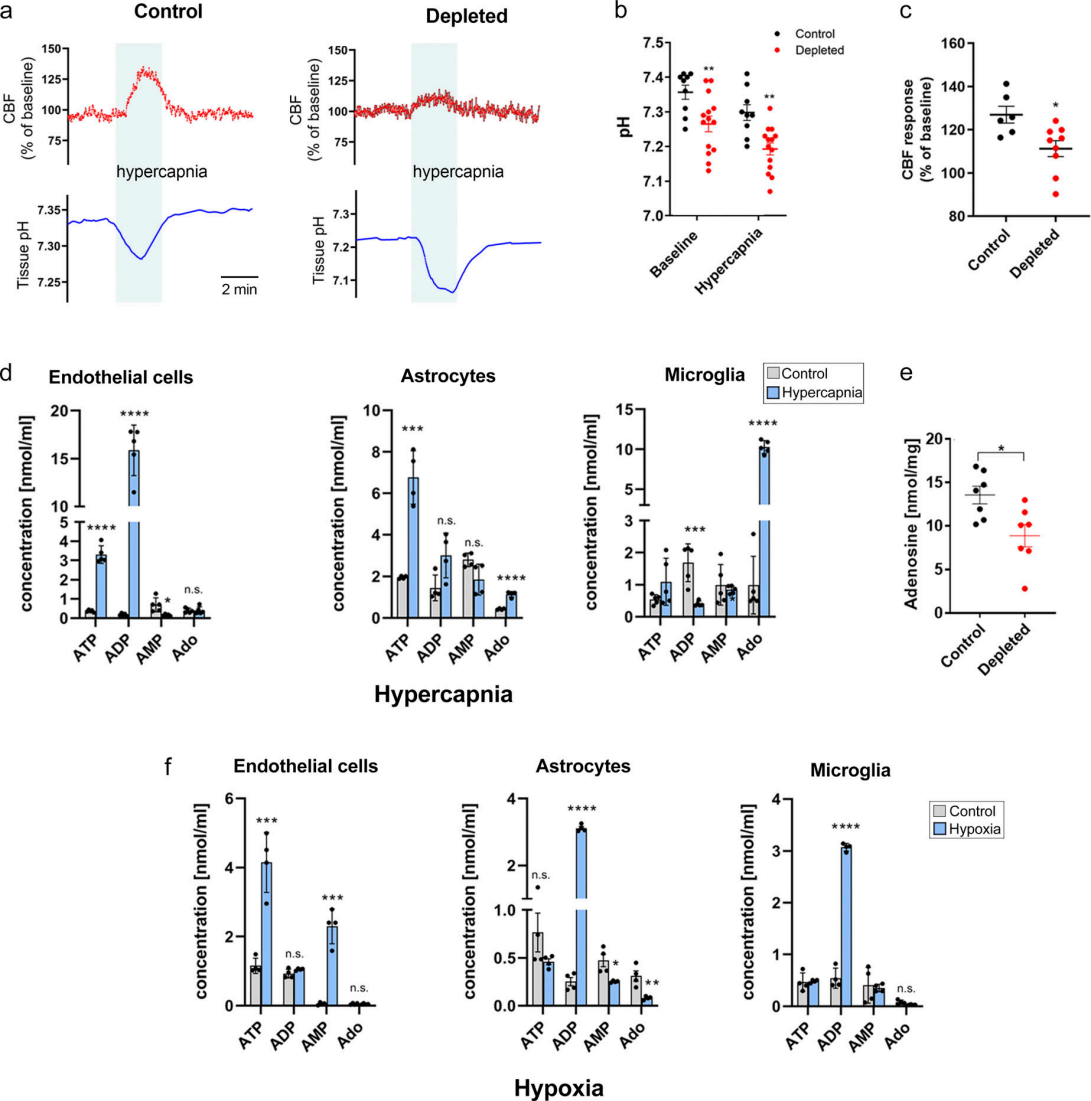
**Stimulus-specific release of purinergic metabolites by NVU cells parallels microglial modulation of brain pH and hypercapnia-induced adenosine production. (a)** CBF by laser Doppler flowmetry and tissue pH by pH-selective electrode were simultaneously assessed during hypercapnic challenge for 2 min. **(b)** Depleted mice show reduced extracellular brain pH. *n* = 10 and *n* = 16 measurements from six control and nine depleted mice; P < 0.0001, two-way ANOVA followed by Sidak’s multiple comparison (**, P = 0.0093, control versus depleted baseline; **, P = 0.0028, control versus depleted hypercapnia). **(c)** CBF response to hypercapnia is reduced in microglia-depleted mice. *n* = 6 control and 9 depleted mice; *, P = 0.012, Mann–Whitney test. **(d)** Effect of hypercapnia on levels of purinergic metabolites (ATP, ADP, AMP, and Ado [adenosine]) in primary endothelial, astrocyte, and microglia cultures as measured by HPLC. Endothelial cells: ATP: ****, P < 0.0001; ADP: ****, P < 0.0001; AMP: *, P = 0.01226, control versus hypercapnia; astrocytes: ATP: ***, P = 0.00029; Ado: ****, P = 0.000057, control versus hypercapnia; microglia; ADP: ***, P = 0.00134; Ado: ****, P < 0.0001, control versus hypercapnia; multiple *t* test. **(e)** Adenosine levels are significantly reduced in the cerebral cortex in the absence of microglia upon hypercapnic challenge. Adenosine was measured by HPLC in cortical brain tissue homogenates. *n* = 7 control and *n* = 7 depleted mice; *, P = 0.0142, unpaired *t* test. **(f)** Effect of hypoxia on levels of purinergic metabolites (ATP, ADP, AMP, and Ado) in primary endothelial, astrocyte, and microglia cultures as measured by HPLC. Endothelial cells: ATP: ***, P = 0.00054; AMP: ***, P = 0.00011, control versus hypercapnia; astrocytes: ADP: ****, P < 0.0001; AMP: *, P = 0.0148; Ado: **, P = 0.0059, control versus hypercapnia; microglia: ADP: ****, P < 0.0001, control versus hypercapnia; multiple *t* test. Data are expressed as mean ± SEM.

Hypercapnia drives vasodilation in the brain mainly through reducing brain pH (Yoon et al., 2012). To further investigate the mechanisms through which microglia shape CBF, cortical blood flow (by laser Doppler) and tissue pH (by pH-selective electrode) were simultaneously assessed during hypercapnia. Surprisingly, baseline brain pH was significantly lower in depleted mice, while the relative amplitude of the hypercapnia-induced negative pH shift was not different in control versus depleted animals (Fig. 6, a and b), suggesting that microglia contribute to modulation of brain pH. As seen previously with LSCI (Fig. 5, e–g), laser Doppler flowmetry confirmed significantly smaller hypercapnia-induced CBF elevation in the absence of microglia (Fig. 6 c).

We next asked whether hypercapnia-induced negative pH shift leads to the production of specific purinergic metabolites (e.g., ATP or ADP) in astrocytes and endothelial cells that may drive microglial process recruitment, as suggested by clustering of microglial P2Y12R at endothelial contact sites near mitochondria (Fig. 1, i and j) and by MicroDREADD^Dq^ experiments (Fig. 4, e and f). Hypercapnic challenge reduced both extracellular and intracellular pH (Fig. S3, c and d) and triggered rapid ATP and adenosine production by cultured astrocytes, while ATP and ADP were produced by endothelial cells (Fig. 6 d), as assessed by HPLC. In contrast, hypoxic challenge resulted in endothelial ATP, but not ADP, production, while astrocytes released mainly ADP (Figs. 6 f and S3 e). Importantly, hypercapnia, but not hypoxia, triggered a robust, 10-fold increase in microglial adenosine production (Fig. 6 d), and hypercapnia-induced adenosine levels were attenuated by microglia depletion in vivo (Fig. 6 e). Collectively, these experiments suggest that hypercapnia and hypoxia lead to rapid production of different purinergic metabolites, with high levels of adenosine, a potent vasodilator, produced by microglia in response to hypercapnic challenge.

### Selective elimination of microglia augments hypoperfusion after common carotid artery occlusion (CCAo)

**Figure 7.**
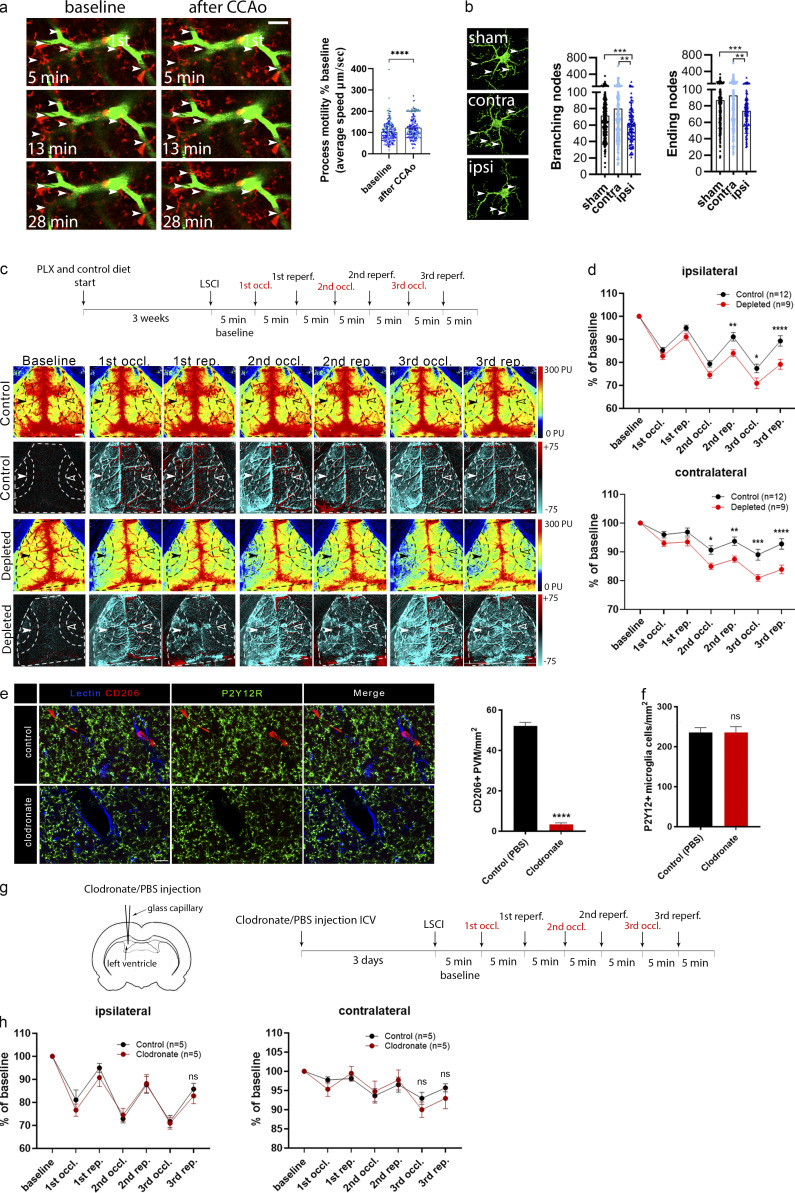
**Adaptation to cortical hypoperfusion is impaired in the absence of microglia. (a)** In vivo two-photon imaging reveals increased microglial process motility (arrowheads) to repeated (3×) CCAo in CX3CR1^tdTomato^ mice (1st, first-order capillary). *n* = 6 mice; ****, P < 0.0001, Mann–Whitney *U* test. Scale bar, 20 μm. **(b)** Automated morphological analysis demonstrates reduced number of branching and ending nodes of microglial processes ipsilaterally in CX3CR1^GFP/+^ mice 24 h after 3× CCAo compared with the contralateral side (contra) and sham animals in the cerebral cortex. Branching/ending nodes of *n* = 386–388 sham, *n* = 197 contralateral (contra), and *n* = 134 ipsilateral (ipsi) cells from *n* = 3 sham and *n* = 3 CCAo mice; ***, P = 0.0008, Kruskal–Wallis test followed by Dunn’s multiple comparisons test (branching nodes: ***, P = 0.0008, sham versus ipsi; **, P = 0.005, contra versus ipsi; ending nodes: ***, P = 0.0007, sham versus ipsi; **, P = 0.0083, contra versus ipsi). **(c)** Representative perfusion (first and third rows), and difference LSCI images (second and fourth rows) showing cortical perfusion changes in response to 3× CCAo (occl.) in control and microglia-depleted mice. Dashed lines indicate the area of quantification in both the ipsilateral (white arrowheads) and contralateral (empty arrowheads) hemisphere as shown in d. Venous sinuses were excluded from the analysis. Scale bar, 1 mm. reperf., reperfusion. **(d)** CBF responses to 3× CCAo are shown as the percentage of baseline. A significant CBF reduction is seen in the absence of microglia in both hemispheres. *n* = 9 control and *n* = 12 depleted mice; ****, P < 0.0001, two-way ANOVA followed by Sidak’s multiple comparison test (ipsilateral second reperfusion [rep.], **, P = 0.0099; third occl., *, P = 0.0270; third rep., ****, P < 0.0001 control versus depleted; contralateral second occl., *, P = 0.0233; second rep., **, P = 0.0052; third occl., ***, P = 0.0001; third rep., ****, P < 0.0001 control versus depleted). **(e)** ICV clodronate administration resulted in the depletion of CD206-positive PVMs but did not affect microglial cells (P2Y12R labeling, green). Blood vessels were visualized using the endothelial marker, tomato lectin (blue). Scale bar, 20 µm. Quantification of the number of PVMs after ICV clodronate liposomes or PBS injection. *n* = 5–5 mice control versus clodronate injected; ****, P < 0.0001, unpaired *t* test with Welch’s correction. **(f)** Quantification of the number of P2Y12-positive microglia cells after ICV clodronate liposomes or PBS injection. *n* = 5–5 mice control versus clodronate injected, unpaired *t* test with Welch’s correction. **(g)** PVMs were eliminated from the brain by ICV liposomal clodronate injection before LSCI measurements. **(h)** No difference in CBF is seen between clodronate-treated and control mice after 3× CCAo. *n* = 5 and 5 mice control versus clodronate injected, two-way ANOVA followed by Sidak’s multiple comparison test. Data are expressed as mean ± SEM. LSCI data have been pooled from two to three independent experiments.

**Figure S4.**
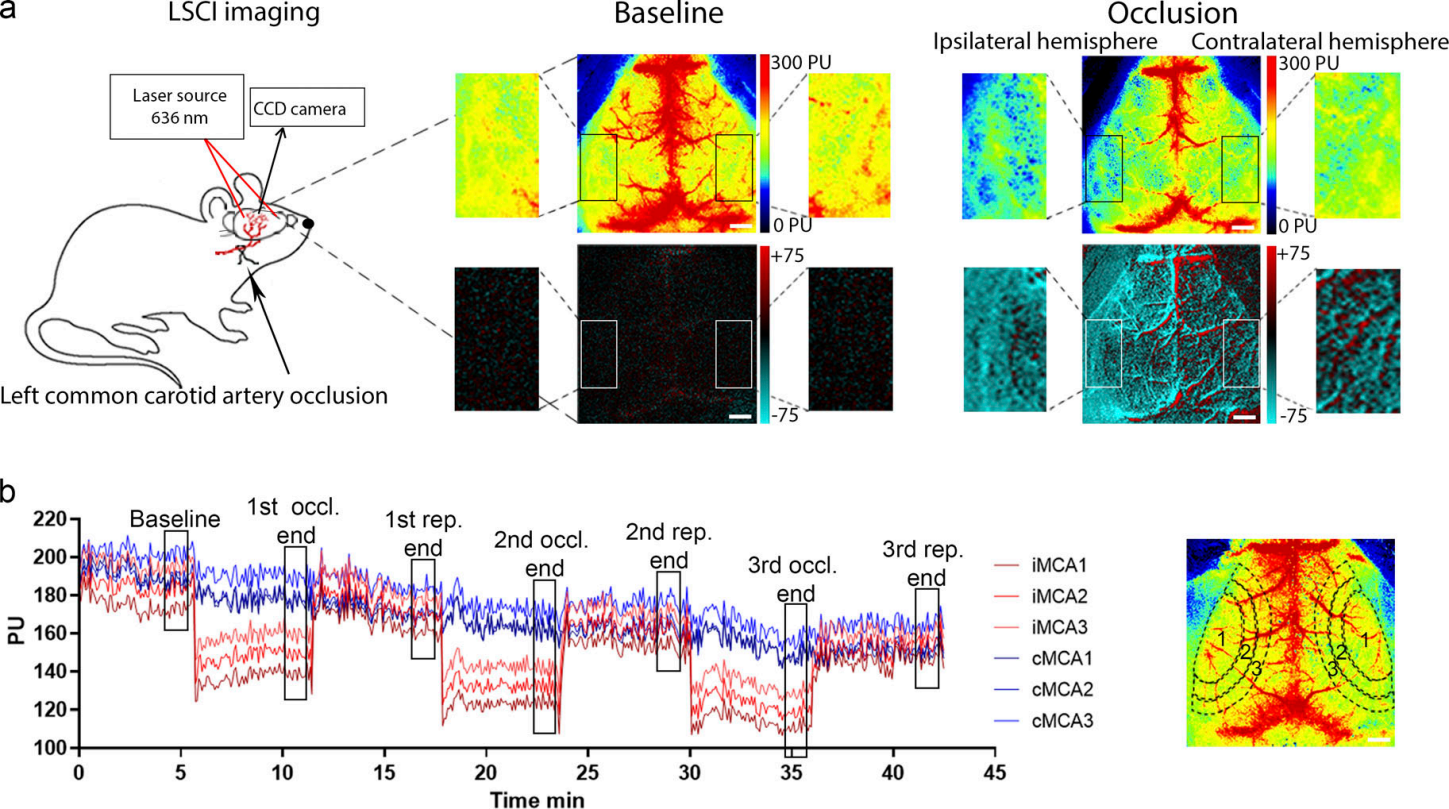
**CBF was measured during transient left CCAo through the intact skull bone by LSCI.** **(a)** Representative perfusion (0–300 PU, on the top of a, and difference images (−75 to +75) on the bottom of a show baseline CBF and perfusion changes during CCA occlusion. Scale bar, 1 mm. **(b)** Representative graph showing the typical kinetics of repeated (3× CCA) occlusions on the areas (MCA1–3 areas) investigated on both hemispheres. ROIs are shown on a representative perfusion image on the right. Black rectangles on the kinetic graph display the sections of curves, which were used for detailed analysis. Scale bar, 1 mm.

Stimulus-specific release of purinergic mediators by different NVU cells suggested that microglial effects on CBF are likely to be important for the maintenance of sufficient cerebral blood perfusion, which is compromised in diverse vascular diseases including stroke, chronic hypoperfusion, or vascular dementia, among others (Iadecola, 2017; Kisler et al., 2017; Wolters et al., 2017). To study the actions of hypoperfusion-primed microglia (Masuda et al., 2011) on subsequent CBF changes, we developed a model by performing repeated transient unilateral CCAo and reperfusion three times (Fig. S4, a and b). Redistribution of blood flow to the ipsilateral cortical circulation requires vasodilation (Polycarpou et al., 2016), and unilateral CCAo does not cause cerebral ischemia (Nishijima et al., 2016; Polycarpou et al., 2016), making this model ideal to study vascular adaptation responses during hypoperfusion in the absence of neuronal injury, which is influenced by microglia manipulation (Szalay et al., 2016). In vivo two-photon imaging revealed rapid microglial process response to CBF reduction, as shown by increased process motility of blood vessel–associated microglia immediately after CCAo (Fig. 7 a). High-resolution automated analysis demonstrated that alterations in microglial process morphology are maintained up to 24 h after CCAo (Fig. 7 b). Importantly, LSCI measurements showed markedly impaired adaptation to reduced cortical perfusion after CCAo in the absence of microglia. This was evidenced by lower baseline-corrected CBF values after 5 min CCAo and subsequent reperfusion for 5 min, which effect gradually increased as CCAo and reperfusion were repeated two more times (P < 0.0001, two-way ANOVA; Fig. 7, c and d; and Fig. S4, a and b). In fact, average CBF values by the third occlusion reached only 79% of baseline in microglia-depleted mice, as opposed to 89% in control mice in the ipsilateral hemisphere (Fig. 7 d and Video 8).

**Video 8.** **Representative LSCI video showing the third occlusion-reperfusion period in microglia-depleted and contol mice.** Difference images display the marked CBF reduction caused by the third occlusion over the second reperfusion period. White ellipses show the area of the cerebral cortex (MCA territory) with significant CBF reduction after CCA occlusion. Note: to reduce the size of the video, a 2-min-long section from the third occlusion and a 3-min-long section from the second reperfusion have been removed. No significant CBF changes were seen during these periods.

Interestingly, the absence of microglia also markedly impaired CBF recovery after repeated CCAo in the contralateral hemisphere (P < 0.0001, two-way ANOVA, Fig. 7 d, and Video 8), indicating that microglial actions are involved in normalizing CBF responses during reperfusion. Impaired CBF recovery was also evident in both hemispheres between the second and the third reperfusions in microglia-depleted mice (2.8-fold larger reduction compared with control mice both ipsilaterally and contralaterally, P = 0.042 and P = 0.048, respectively, unpaired *t* test). Selective elimination of PVMs by intracerebroventricular (ICV) administration of clodronate without an effect on resident microglia (Fig. 7, e and f) did not influence blood flow responses after repeated CCAo (Fig. 7, g and h), suggesting that microglia sense and influence CBF changes differently in this model of hypoperfusion than other brain macrophages.

### The effect of microglial actions on CBF is mediated via P2Y12R signaling during hypoperfusion

**Figure 8.**
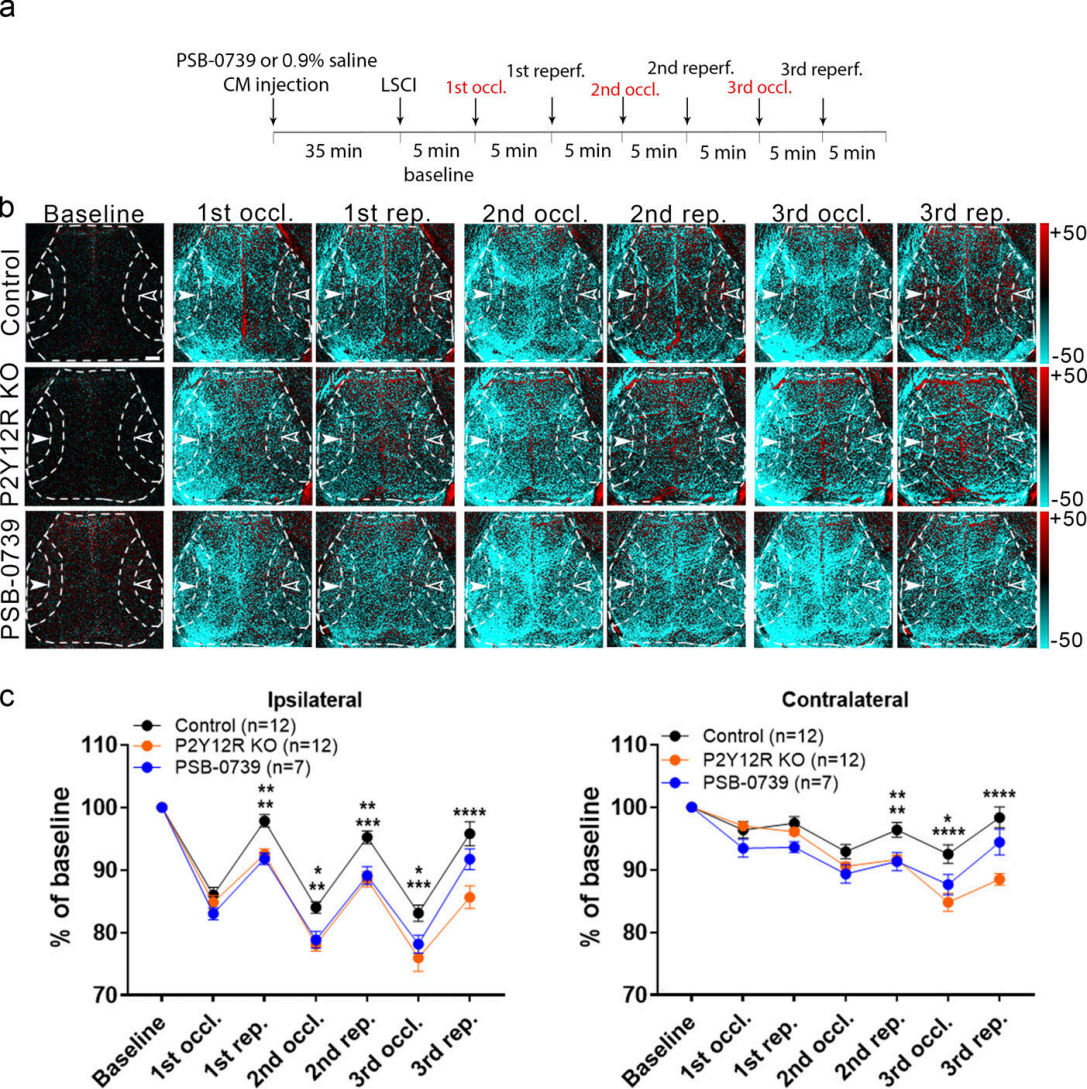
**Microglial actions on CBF require P2Y12R signaling. (a)** Outline of the experimental 3× CCAo protocol (occl., occlusion; reperf., reperfusion). **(b)** Representative difference images show altered perfusion in both hemispheres in response to 3× CCAo in P2Y12R KO and PSB0739-injected mice compared with controls. Dashed lines show the MCA2 area both in the ipsilateral (white arrowheads) and in the contralateral hemisphere (empty arrowheads) corresponding to the quantitative analysis shown in c. Scale bar, 1 mm. rep., reperfusion. **(c)** A significant impairment in adaptation to hypoperfusion is seen both in the ipsilateral and contralateral hemispheres of P2Y12R KO mice and PSB0739-injected mice compared with controls. *n* = 12 control, *n* = 12 P2Y12R KO, *n* = 7 PSB0739-injected mice; ****, P < 0.0001, two-way ANOVA followed by Tukey’s multiple comparison test (ipsilateral first rep., **, P = 0.0042 control versus P2Y12R KO; **, P = 0.0057 control versus PSB-0739; second occl., **, P = 0.0013 control versus P2Y12R KO; *, P = 0.0214 control versus PSB-0739; second rep., ***, P = 0.0002 control versus P2Y12R KO; **, P = 0.0049 control versus PSB-0739; third occl., ***, P = 0.0001 control versus P2Y12R KO; *, P = 0.0302 control versus PSB-0739; third rep., ****, P < 0.0001 control versus P2Y12R KO; contralateral second rep., **, P = 0.004 control versus P2Y12R KO; **, P = 0.0096 control versus PSB-0739; third occl., ****, P < 0.0001 control versus P2Y12R KO; *, P = 0.0133 control versus PSB-0739; third rep., ****, P < 0.0001 control versus P2Y12R KO). Data are expressed as mean ± SEM. LSCI data have been pooled from two to three independent experiments.

Our HPLC studies demonstrated that both ADP and ATP are released rapidly by NVU cells in response to hypercapnia and hypoxia. ADP, which can rapidly form upon ATP hydrolysis by ectoATPases, is the main ligand for microglial P2Y12R expressed by microglial processes (Cserep et al., 2019), among other cells. To this end, we tested whether an inhibition of microglial P2Y12R using either genetic deletion of P2Y12R or acute pharmacological blockade by PSB0739 injected into the cisterna magna (Cserep et al., 2019) alters CBF responses after repeated CCAo. Importantly, we found that blood flow recovery was markedly impaired after both genetic and pharmacological P2Y12R blockade in the ipsilateral and contralateral hemispheres (Fig. 8, a–c), similar to that seen in microglia depletion studies (Fig. 7). Thus, these results collectively suggest that both microglia and microglial P2Y12R are essential for normalizing CBF responses during adaptation to reduced cortical perfusion after CCAo.

## Discussion

Here, we identify microglia as a novel cell type modulating blood flow in the brain. Using three different experimental models, we show that the presence of functional microglia is essential to maintain optimal CBF responses to physiological neuronal activity and hypercapnia and during cerebrovascular adaptation to reduced cortical perfusion after CCAo. These actions are dependent on microglial P2Y12R signaling, clearly discriminating microglial responses from those mediated by PVMs or other brain macrophages (Prinz et al., 2017).

While microglia produce several vasoactive or inflammatory mediators, including IL-1β, TNF-α, NO, PGE2, or ROS (Wolf et al., 2017), that may modulate cerebral perfusion (Iadecola, 2017; Zhao et al., 2018), the potential contribution of microglia to CBF has been largely neglected to date. Instead, research has focused on their role in BBB function, extravasation of leukocytes, and angiogenesis from embryonic stages into adulthood (Dudvarski Stankovic et al., 2016). Because microglial cell bodies are located in the brain parenchyma, while the endothelial basal lamina is surrounded by a second, glial basement membrane (Engelhardt and Sorokin, 2009), we first asked whether a direct contact between microglia and endothelial cells exists in the adult neocortex. We found that microglia dynamically contact different levels of the vascular tree in vivo and establish direct, purinergic contacts with endothelial cells, periarterial smooth muscle cells, pericytes, and astrocytes in both the mouse and the human brain, which regulate blood flow. These observations suggested that purinergic mediators, such as ATP or ADP may be released from NVU cells to recruit P2Y12R-positive microglial processes during vascular adaptation responses or perfusion changes, even under physiological conditions.

To investigate whether microglia could influence CBF responses to physiological neuronal activity, we turned to the widely used whisker stimulation model. Neurovascular coupling is a dynamic functional change in CBF in response to local neuronal activity, which involves different cell types within the NVU, including astrocytes, vascular smooth muscle cells, pericytes, and endothelial cells (Iadecola, 2017; Kisler et al., 2017). However, a role for microglia has not been previously established. During functional hyperemia, dilation of arterioles propagates at high speed in a retrograde direction to upstream arteries, including branches of pial arteries, with both arteriolar and capillary dilation playing a role in increased O_2_ delivery (Kisler et al., 2017). Our LSCI and fUS studies revealed significantly smaller CBF response to whisker stimulation in the barrel cortex in the absence of microglia, or microglial P2Y12R, which was not explained by altered neuronal responses in the barrel cortex as assessed by in vivo electrophysiology or two-photon calcium imaging. While neuronal activity during hypercapnia was not different between control, P2Y12R KO, and microglia-depleted mice either, it remains to be investigated whether microglia-dependent effects may also influence CBF through changing baseline activity of neurons that control blood flow in the brain (Badimon et al., 2020; Cserep et al., 2019; Kisler et al., 2017). To test the specificity of the microglial actions observed, we developed a mouse model allowing selective chemogenetic targeting of microglia in real time in vivo, which disrupts normal microglial process dynamics and renders depolarized cells less responsive to ambient ATP. Smaller CBF responses to whisker stimulation upon chemogenetically induced microglial dysfunction suggest that sustained microglial sensing of purine metabolites and directed process recruitment are required to modulate functional hyperemia in the cerebral microcirculation. These experiments also indicate that even temporary impairment in the dynamic communication between microglial processes and the vasculature could have marked impact on CBF and tentatively on other vascular responses, which has broad implications to any pathological conditions that are associated with altered microglial phenotypes.

We next tested whether microglia-mediated mechanisms influence vascular responses to hypercapnia. Hypercapnia induces vasodilation via complex actions that involve NO release from the endothelium, relaxation of smooth muscle cells and pericytes, release of astrocytic prostaglandin E2, and other processes (Faraci et al., 2019; Hamilton et al., 2010; Howarth et al., 2017; Meng and Gelb, 2015; Yoon et al., 2012). Importantly, while perivascular microglial processes rapidly responded to hypercapnia with calcium pulses and generation of new phylopodia, the absence of microglia markedly inhibited increases of CBF (as demonstrated independently by both LSCI and laser Doppler flowmetry) and vasodilation (as shown by in vivo two-photon imaging). This was independent of arterial blood pH, pO_2_, and pCO_2_ levels, which were not different in microglia-depleted mice. Surprisingly, we found that absence of microglia reduced brain pH, while microglia rapidly produced adenosine in response to hypercapnia. Supporting this, recent findings showed that microglia represent a key source of adenosine in the brain, which modulates neuronal responses at synapses (Badimon et al., 2020). Hypercapnia drives vasodilation mainly via reduced extracellular pH, which is a major regulator of cerebrovascular reactivity and acts directly on cerebrovascular smooth muscle cells to cause relaxation, mediating the effects of increased CO_2_ levels (Yoon et al., 2012). Microglial P2Y12R-mediated Ca^2+^ signaling, migration, and cytokine production are also pH dependent (Jin et al., 2014; Langfelder et al., 2015). Because adenosine is a potent vasodilator in the cerebral circulation (Pelligrino et al., 2011), we suggest that lower brain pH in the absence of microglia may partially compensate for the loss of microglial vasoactive mediators, with a net effect of reduced vasodilation during different vascular adaptation responses. It should be investigated in future studies whether microglial loss or dysfunction could induce compensatory actions in other NVU cells, such as promoting adenosine production by astrocytes (MacVicar and Newman, 2015).

To further investigate the mechanisms through which microglial P2Y12R may modulate vascular responses, we investigated the possible links with NO, a key mediator of vasodilation (Attwell et al., 2010; Iadecola, 2017; Toda et al., 2009). The observation that the absence of microglia and NO blockade by L-NAME had an additive effect to reduce the coupling response upon somatosensory stimulation, strongly suggests that P2Y12R-positive microglia regulate the CBF response to somatosensory stimulation through signaling mechanisms that are, at least in part, additional to NO-mediated vasodilation. This may have broad physiological and pathological consequences given the complexity of CBF regulation in health and disease (Attwell et al., 2010; Iadecola, 2017; Kisler et al., 2017; Toda et al., 2009).

These conclusions are extended further by our hypercapnia studies. NO functions, including vasodilation, are mediated by cGMP synthesized through soluble guanylyl cyclase, a heme-containing enzyme, which is directly activated by NO (Toda et al., 2009). Rapid response of microglia to hypercapnia as demonstrated by calcium fluctuations and generation of perivascular phylopodia, which was P2Y12R dependent in vivo, together with inhibition of hypercapnia-induced cGMP by P2Y12R blockade in CD13-positive, contractile elements (smooth muscle cells and pericytes) ex vivo, suggest that contacting microglial processes may interfere with vasodilation via tentatively different cell types and mediators, which may include NO and adenosine. It should be noted that hypercapnia also increased cGMP in CD13-positive profiles in vivo, but the extent of this response was heterogeneous, most likely due to the difficulties with precise timing of tissue collection and the rapid hydrolysis of cGMP by phosphodiesterases, which we were able to block effectively in acute brain slices (Szabadits et al., 2011). It will also need to be investigated further in future studies how microglial modulation of NO actions could interact with the production of adenosine or other vasoactive mediators by microglia and other cells in the NVU.

We finally asked whether microglia sense and respond to cerebral hypoperfusion. CBF is controlled by feed-forward and feedback mechanisms that maintain or re-establish optimal oxygen and nutrient supply of neurons in case disturbances of the cardiovascular system occur (Colonna and Butovsky, 2017). Adaptation to reduced cerebral perfusion requires vasodilation (Zaharchuk et al., 1999). Unilateral CCAo is an established model of cerebrovascular adaptation to the reduction of perfusion, which is mediated primarily by the activation of feedback pathways through the collateral circulation (Polycarpou et al., 2016), while it does not induce neuronal death or BBB injury in rodents (Nishijima et al., 2016; Polycarpou et al., 2016). Since the cell types in the NVU contacted by microglia regulate CBF (Attwell et al., 2010; Iadecola, 2017; Kisler et al., 2017), we argued that microglia primed by hypoperfusion during the first occlusion would interfere with subsequent vascular adaptation responses, and hence elimination of microglia may alter CBF after repeated CCAo. As supported by previous results showing that microglial process responses around microvessels change proportionally to the level of CBF reduction (Masuda et al., 2011), we found that microglial processes rapidly respond to CCAo. Importantly, absence of microglia and both genetic and pharmacological blockade of microglial P2Y12R resulted in impaired adaptation to reduced cortical perfusion during repeated CCAo, which strengthens their different roles compared with P2Y12R-negative PVMs (Cserep et al., 2019; Fekete et al., 2018), as also confirmed by the lack of an effect of PVM depletion.

The importance of ATP signaling in the vasculature has been demonstrated under both homeostatic and pathological conditions (Lohman et al., 2012). Microglial processes are recruited to sites of ATP release via P2Y12R, which primarily sense ADP produced by ATP hydrolysis or cleavage by NTPDase1 expressed on the microglial membrane, among other cells (Cserep et al., 2019; Davalos et al., 2005; Haynes et al., 2006). Our electron tomography studies revealed an accumulation of P2Y12R on microglial processes contacting endothelial cells in the vicinity of endothelial mitochondria, where ATP release may recruit microglial processes to the vasculature in response to CBF changes (Lohman et al., 2012). Similar interactions are seen at somatic purinergic junctions (Cserep et al., 2019), through which microglia sense neuronal mitochondrial activity and modulate neuronal responses via purinergic signaling. ATP derived from astrocytes is also known to constrict vascular smooth muscle cells and regulate blood flow (Kisler et al., 2017). Importantly, our HPLC studies demonstrated that endothelial cells and astrocytes release different purinergic metabolites in response to hypoxia and hypercapnia, both of which occur during hypoperfusion. While we found rapid alterations in microglia–endothelium and microglia–astrocyte interactions after CCAo and hypercapnia, hypoxia and hypercapnia also triggered different purinergic responses in microglia. Although the mechanisms through which different purinergic mediators are released in the NVU remain to be explored, pannexin-1 (PANX1) channels are likely to be involved, which is also suggested by reduced hypercapnia-induced CBF responses in PANX1 KO mice (Bisht et al., 2021). Thus, cell- and stimulus-specific production of vasoactive metabolites may provide means for different vascular adaptation responses, during which microglia may alter CBF via actions on different cell types in the NVU or protect against mild hypoxia-induced vascular leakage (Halder and Milner, 2019). Our in vivo two-photon imaging data also indicate that individual microglial processes may perform functionally distinct tasks to influence vascular (and other) responses in given microdomains, which is largely supported by the high level of functional autonomy of calcium signaling in microglial processes (Umpierre et al., 2020).

Because all experimental models have their limitations, we made efforts to use alternative approaches wherever possible during these complex studies. For example, prolonged (7-wk-long) treatment with PLX5622 has been found to affect the choroidal vasculature and alter angiogenic growth (Yang et al., 2020). However, the structural/cellular integrity of blood vessels was not found to be disturbed in the neocortex after 3 wk of depletion in the present study, as also seen earlier (Elmore et al., 2014; Szalay et al., 2016). This is also confirmed by our [^99m^Tc]-HMPAO SPECT and [^18^F]-FDG PET measurements, which are widely used noninvasive methods to assess regional perfusion and glucose metabolism changes, respectively (Apostolova et al., 2012; Tai and Piccini, 2004). Another possible confounder could be that CX3CR1 (used as a promoter in the CX3CR1^CreERT2^ driver line to generate MicroDREADD^Dq^ mice) may also be expressed by other brain macrophages apart from microglia (Kim et al., 2021). It is theoretically possible that in addition to microglia, other brain myeloid cells such as meningeal macrophages or PVMs could have contributed to shaping vascular responses in the present study. However, in line with the very high specific recombination rate of microglia in CX3CR1tdTomato microglia reporter mice and MicroDREADD^Dq^ mice, we demonstrated that all parenchymal CX3CR1-positive cells were P2Y12R-positive microglia. Importantly, the contribution of microglia to CBF regulation has been confirmed with a number of independent strategies in all three experimental models, including pharmacological and genetic blockade of P2Y12R, which is specific for microglia in the central nervous system (Cserep et al., 2019). Effective blockade of microglial P2Y12R by PSB0739 injected into the cisterna magna has also been characterized in detail in our previous study (Cserep et al., 2019).

### Clinical significance

We believe that the implication of these data is far-reaching. Altered microglial activity and impaired CBF or neurovascular coupling precede symptom onset in common brain pathologies such as Alzheimer’s disease, Lewy body dementia, idiopathic Parkinson’s disease, chronic hypoperfusion, and amyloid angiopathy (Attwell et al., 2010; Iadecola, 2017; Kisler et al., 2017; Wolf et al., 2017). Thus, dysfunction of microglia could contribute to disease pathophysiology by modulating CBF via endothelial cells or other cells in the NVU. Interestingly, homozygous missense mutations of TREM2 (a microglial receptor) are linked with increased risk of dementia, while Trem2 p.T66M knock-in mice display an age-dependent reduction in microglial activity, CBF, and brain glucose metabolism (Kleinberger et al., 2017). In patients with risk factors for stroke, carotid stenosis, aneurysm, hypertension, chronic vascular inflammation, or transient ischemic attack, altered microglial activity may impact clinical outcome merely via modulating cerebral perfusion or adaptation to reduced perfusion. As such, microglia could also contribute to ischemic preconditioning, vasospasm after subarachnoid hemorrhage, or the “no reflow phenomenon” after cerebral ischemia (Kloner et al., 2018), while microglial surveillance is likely to be disturbed during hypoxia or ischemia, as evidenced in the developing brain (Eyo and Dailey, 2012; Masuda et al., 2011).

In conclusion, our data demonstrate that microglia should be considered an important modulatory cell type involved in physiological and pathological alterations of CBF. Understanding their actions may facilitate the discovery of novel treatment opportunities in common neurological disorders.

## Materials and methods

### Mice

Experiments were carried out on 11–17-wk-old C57BL/6J (RRID:IMSR_JAX:000664); P2Y12R^−/−^ (B6;129-P2ry12^tm1Dgen^/H P2Y12R KO); CX3CR1^GFP/+^ (RRID:IMSR_JAX:005582), CX3CR1^GFP/+^/P2Y12^−/−^, CX3CR1^GFP/GFP^, CX3CR1^tdTomato^, and Thy1-GCaMP6s (C57BL/6J-Tg[Thy1-GCaMP6s]GP4.12Dkim/J, RRID:IMSR_JAX:025776; Dana et al., 2014); MicroDREADD^Dq^, CX3CR1^CGaMP5g–tdTomato^ (RRID:IMSR_JAX:024477); and MicroDREADD^Dq^ × CGaMP5g–tdTomato mice (all on C57BL/6J background). Mice were kept in a 12-h dark/light cycle environment, under controlled temperature and humidity, with food and water ad libitum. All experimental procedures were in accordance with the guidelines set by the European Communities Council Directive (86/609 EEC) and the Hungarian Act of Animal Care and Experimentation (1998; XXVIII, Sect. 243/1998), approved by the Animal Care and Experimentation Committee of the Institute of Experimental Medicine and the Government Office of Pest County Department of Food Chain Safety, Veterinary Office, Plant Protection and Soil Conservation Budapest, Hungary, under the numbers PE/EA/1021-7/2019 and PE/EA/673-7/2019, and the Department of Food Chain Safety and Animal Health Directorate of Csongrád County, Hungary. Experiments were performed according to EU Directive 2010/63/EU on the protection of animals used for scientific purposes and are reported in compliance with the ARRIVE guidelines.

### Generation of CX3CR1^tdTomato^, MicroDREADD^Dq^, MicroDREADD^Dq^ × CGaMP5g–tdTomato, and CX3CR1^CGaMP5g–tdTomato^ mice

CX3CR1^tdTomato^, MicroDREADD^Dq^, CX3CR1^CGaMP5g–tdTomato^, and MicroDREADD^Dq^ × CGaMP5g–tdTomato mice were generated by crossing TMX-inducible CX3CR1^CreERT2^ mice (B6.129P2[C]-CX3CR1^tm2.1[cre/ERT2]Jung/J^, RRID:IMSR_JAX:020940; Yona et al., 2013) with a mouse line expressing Cre-dependent tdTomato (B6;129S6-Gt[ROSA]26Sortm9[CAG-tdTomato]Hze/J, RRID:IMSR_JAX:007905), hM3Dq DREADD (B6N;129-Tg[CAG-CHRM3*,-mCitrine]1Ute/J (Zhu et al., 2016), RRID:IMSR_JAX:026220), or CGaMP5g–tdTomato (B6;129S6-*Polr2a*^*Tn[pb-CAG-GCaMP5g,–tdTomato]Tvrd*^/J; Gee et al., 2014; RRID:IMSR_JAX:024477). To induce tdTomato, hM3Dq DREADD, or CGaMP5g–tdTomato expression in microglia, Cre recombinase activity was induced by two i.p. injections of TMX (2 mg/100 μl, dissolved in corn oil; #T5648; Sigma-Aldrich), 48 h apart in 3–4-wk-old male mice, shortly after weaning. 4 wk after TMX induction, 95.3% of microglia expressed hM3Dq receptors, as confirmed by anti-GFP (goat anti-GFP antibody, 1:300; #600-101-215; Rockland) and anti-P2Y12R immunostaining, to detect mCitrine and microglia, respectively (Zhu et al., 2016). Using CX3CR1^CreERT2^ mice, microglia show constant Cre-dependent expression, while most peripheral macrophages/monocytes expressing CX3CR1 are replaced by the end of the fourth week after TMX induction, owing to their rapid turnover (Yona et al., 2013). Therefore, all experiments were carried out at 11 and 12 wk of age. Microglial responses were modulated in real time either by i.p. CNO (0.5 mg/kg; #4936; Bio-Techne Corp.) or i.p. deschloroclozapine (1 µg/kg; #HB9126; HelloBio) administration, via the activation of hM3Dq DREADD (Alexander et al., 2009).

### Characterization of CX3CR1^tdTomato^ microglia reporter mice

4 wk after TMX induction, virtually 100% of microglia in the cerebral cortex expressed tdTomato, as assessed by P2Y12 and Iba1 immunostaining. In the whole population, 94% of Tomato-positive cells coexpressed both P2Y12 and Iba1, 2.4% of the cells were only Iba1-positive, and 3.6% of tdTomato cells did not express either Iba1 or P2Y12. tdTomato-positive cell bodies and processes were analyzed in the somatosensory cortex on high-resolution CLSM stacks (CFI Plan Apochromat VC 60XH oil-immersion objective, 0.1 µm/pixel, Z-step: 2 µm) with homogeneous sampling (Fig. S1 h). Cell nuclei were identified by DAPI.

### In vivo two-photon imaging

Cranial window preparation in CX3CR1^GFP/+^, CX3CR1^tdTomato^, CX3CR1^GFP/+^ × P2Y12^−/−^, Thy1-GCaMP6s CX3CR1^CGaMP5g–tdTomato^, and MicroDREADD^Dq^ × CGaMP5g–tdTomato mice was performed above the primary somatosensory or the barrel cortex. Blood vessels were labeled with either Rhodamine B-dextran or FITC-dextran (70,000 mol wt). 3 wk after cranial window surgery, microglia–vascular interactions in response to 3× CCAo or hypercapnia (ketamine-medetomidine anesthesia, i.p. 30–0.1 mg/kg) and neuronal intracellular Ca^2+^ ([Ca^2+^]_i_) in response to whisker stimulations (in ketamine-medetomidine anesthesia) were imaged in body temperature–controlled animals. For microglia process motility measurements in response to 3× CCAo, the galvo-scanning light path with 16× water-immersion objective (Nikon CFI75 LWD 16× W, NA 0.8) was used to acquire four-image Z-stacks with 8.5-µm step size, 150–200 µm below the dura, at 500 × 500-pixel resolution. For measuring vascular responses to hypercapnia, after obtaining 1 min at baseline, a 2-min hypercapnic episode (inhaling a 10% CO_2_-containing air mixture) and 1-min posthypercapnic period were imaged with the resonant light path at 32.7521 Hz. For in vivo [Ca^2+^]_i_ imaging in control and microglia-depleted Thy1-GCaMP6s mice, the right whiskers were stimulated, and neuronal [Ca^2+^]_i_ transients were imaged in the left barrel cortex at 920-nm wavelength using the resonant scanner at 180–250-µm depth below the dura, under ketamine-medetomidine sedation. The stimulation protocol consisted of 5-Hz square pulses for 15 s, repeated 6 times with 40-s intervals. Measurements were performed at 32.48 Hz using a Nikon 16× water-immersion objective. Imaging was performed by a Femto2D-DualScanhead microscope (Femtonics) coupled with a Chameleon Discovery laser (Coherent). Data acquisition was performed with MESc software (v.3.5.6.9395SLE; Femtonics), and data were analyzed in MES software (v.5.3560; Femtonics).

### Tissue processing and immunostaining

Under terminal (ketamine–xylazine) anesthesia, mice were transcardially perfused with 4% paraformaldehyde, and brains were dissected. Brain samples were postfixed and cryoprotected for 24 h, and 25-µm-thick coronal sections were cut using a sledge microtome (Leica). Immunostaining was performed on free-floating brain sections, blocked with 5% normal donkey serum (Jackson ImmunoResearch). The following primary antibodies were used: rabbit anti-P2Y12R (1:500; #55043AS; AnaSpec), chicken anti-GFP-tag (1:500; #A10262; Invitrogen), goat anti-GFP (1:300; #600-101-215; Rockland), rat anti-CD206 (1:200; #MCA2235; AbD Serotec), and biotinylated tomato lectin (1:100; #B-1175; Vectorlabs). After washing, sections were incubated with the corresponding secondary antibodies (from Jackson ImmunoResearch): donkey anti-rabbit A647 (1:500; #711-605-152), donkey anti-chicken A488 (1:500; #703-546-155), donkey anti-goat A488 (1:500; #705-546-147), donkey anti-rat A594 (1:500; # 712-586-153), and streptavidin DyL405 (1:500; #016-470-084). Slices were mounted with Fluoromount-G (SouthernBiotech) or Aqua-Poly/Mount (Polysciences). For high-resolution CLSM and EM assessments, 50-µm-thick vibratome sections were washed in PB and TBS, followed by blocking with 1% human serum albumin (HSA). Sections were then incubated in different mixtures of primary antibodies: rabbit anti-P2Y12R (1:500; #55043AS; AnaSpec), chicken anti-GFAP (1:500; #173 006; Synaptic Systems), goat anti-PDGFR-β (1:500; #AF1042; R&D Systems), rat anti-CD206 (1:200; #MCA2235; AbD Serotec), rat anti-PECAM-1 (1:500; #102 501; BioLegend), mouse anti-αSMA (1:250; #ab7817; Abcam), guinea pig anti-AQP4 (1:500; #429 004; Synaptic Systems), mouse anti-TOM20 (1:500; #H00009804-M01; Abnova), guinea pig anti-Iba1 (1:500; #234 004; Synaptic Systems), mouse anti-Kv2.1 (1:500; #75-014; NeuroMab), and biotinylated tomato lectin (1:100; #B-1175; Vectorlabs). After washing in TBS, sections were incubated in the corresponding mixtures of secondary antibodies (from Jackson ImmunoResearch): donkey anti-chicken DyLight405 (1:500; #703-474-155), donkey anti-chicken A488 (1:500; #703-546-155), donkey anti-chicken A647 (1:500; #703-606-155), donkey anti-rabbit A647 (1:500; #711-605-152), donkey anti-rabbit A488 (1:500; #A21206; Invitrogen), donkey anti-rat A594 (1:500; #A21209; Invitrogen), donkey anti-rat A647 (1:500; #712-606-153), donkey anti-mouse A594 (1:500; #A21203; Invitrogen), donkey anti-mouse A647 (1:500; #715-605-150), donkey anti–guinea pig DyLight405 (1:500; #706-476-148), donkey anti–guinea pig A594 (1:500; #706-586-148), donkey anti–guinea pig A647 (1:500; #706-606-148), streptavidin DyL405 (1:500; #016-470-084), and streptavidin A594 (1:500; #S11227; Invitrogen). Incubation was followed by washing in TBS and PB, then sections were mounted on glass slides with Aqua-Poly/Mount (Polysciences). Immunofluorescence was analyzed using a Nikon Eclipse Ti-E inverted microscope (Nikon Instruments), with a CFI Plan Apochromat VC 60× oil-immersion objective (NA 1.4) or a Plan Apochromat VC 20× objective (NA 0.75) and an A1R laser confocal system. The following lasers were used: 405, 488, 561, and 647 nm (CVI Melles Griot). Scanning was done in line serial mode. Image stacks were obtained with NIS-Elements AR 5.00.00 software.

### Pre-embedding immuno-EM

After extensive washes in PB and TBS (pH 7.4), vibratome sections were blocked in 1% HSA. Then, they were incubated with rabbit anti-P2Y12R (1:500; #55043AS; AnaSpec) alone or mixed with mouse anti-GFAP (1:1,000; #G3893; Sigma-Aldrich) in TBS for 2–3 days. After several washes, sections were incubated in blocking solution (Gel-BS) containing 0.2% cold water fish skin gelatin and 0.5% HSA for 1 h. Next, sections were incubated with 1.4 nm gold-conjugated goat anti-rabbit Fab-fragment (1:200; #2004; Nanoprobes) alone or mixed with biotinylated donkey anti-mouse (1:500; #715-065-150; Jackson ImmunoResearch) antibodies diluted in Gel-BS overnight. After extensive washes, sections were treated with 2% glutaraldehyde for 15 min to fix the gold particles into the tissue. For the combined immunogold-immunoperoxidase reactions, this was followed by an incubation in avidin–biotinylated HRP complex (Vectastain Elite ABC kit; 1:300; Vector Laboratories) for 3 h at room temperature (RT) or overnight at 4°C. The immunoperoxidase reaction was developed using 3,3-diaminobenzidine (Sigma-Aldrich) as chromogen. To develop the immunogold reaction, sections were incubated in silver enhancement solution (SE-EM; Aurion) for 40–60 min at RT. The sections were then treated with 0.5% OsO_4_ in PB, at RT, dehydrated in ascending alcohol series and in acetonitrile, and embedded in Durcupan (ACM; Fluka). During dehydration, sections were treated with 1% uranyl acetate in 70% ethanol for 20 min. For EM analysis, tissue samples from the somatosensory cortex (S1) were glued onto Durcupan blocks. Consecutive 70-nm-thick (for conventional EM analysis) or 150-nm-thick (for electron tomography) sections were cut using an ultramicrotome (Leica EM UC6) and picked up on Formvar-coated single-slot grids. Ultrathin sections for conventional EM analysis were examined in a Hitachi H-7100 electron microscope equipped with a Veleta charge-coupled device (CCD) camera (Olympus Soft Imaging Solutions). 150-nm-thick electron tomography sections were examined in FEI Tecnai Spirit G2 BioTwin TEM equipped with an Eagle 4k camera.

### Electron tomography and analysis

Before electron tomography, serial sections on single-slot copper grids were photographed with a Hitachi H-7100 electron microscope and a Veleta CCD camera. Serial sections were examined at lower magnification, and P2Y12R-positive microglial processes contacting the vasculature were selected. After this, grids were put on drops of 10% HSA in TBS for 10 min, dipped in distilled water (DW), put on drops of 10-nm gold–conjugated Protein-A (#AC-10-05; Cytodiagnostics) in DW (1:3), and washed in DW. Electron tomography was performed using a Tecnai T12 BioTwin electron microscope equipped with a computer-controlled precision stage (FEI; CompuStage). Acquisition was controlled via Xplore3D software (FEI). Regions of interest (ROIs) were pre-illuminated for 4–6 min to prevent further shrinkage. Dual-axis tilt series were collected at 2°-increment steps between −65 and +65° at 120 kV acceleration voltage and 23,000× magnification with −1.6 to −2 µm objective lens defocus. Reconstruction was performed using the IMOD software package (Kremer et al., 1996). Isotropic voxel size was 0.49 nm in the reconstructed volumes. After combining the reconstructed tomograms from the two axes, the nonlinear anisotropic diffusion filtering algorithm was applied to the volumes. Segmentation of different profiles was performed on the virtual sections using 3Dmod software.

### Postmortem human brain samples

Postmortem human brain tissue was obtained from one 60-yr-old female, one 73-yr-old male, and one 27-yr-old male, without any known neurological disease as also confirmed by neuropathological examination (ETT TUKEB 31443/2011/EKU [518/PI/11]). Informed consent was obtained for the use of brain tissue and for access to medical records for research purposes. Tissue was obtained and used in a manner compliant with the Declaration of Helsinki. Brains of patients who died of nonneurological diseases were removed 4–5 h after death (Table S1). The internal carotid and the vertebral arteries were cannulated, and the brain was perfused first with heparin containing physiological saline, followed by a fixative solution containing 4% paraformaldehyde, 0.05% glutaraldehyde, and 0.2% picric acid (vol/vol) in PB. The hippocampus was removed from the brain after perfusion and was postfixed overnight in the same fixative solution, except that glutaraldehyde was excluded. Blocks were dissected, and 50-µm-thick sections were prepared on a vibratome (VT1200S; Leica).

### LSCI

CBF was measured by a PeriCam PSI High Resolution LSCI system (Perimed AB) at 21 frames/s frequency in a 10 × 10-mm field of view. Perfusion responses were expressed as a percentage of baseline CBF. Uniformly, a 1-min-long baseline was set in all experiments, registered at the beginning of the measurements. Three adjacent ROIs were placed (denoted as MCA1-3) over the middle cerebral artery (MCA) territory both to the ipsilateral and to the contralateral hemispheres to assess microglia-mediated effects on gradual perfusion changes ranging from the MCA core region to the midline. During whisker stimulation, ROIs were placed over the contralateral barrel cortex, while during hypercapnic challenge, ROIs were placed over the left and right hemispheres excluding venous sinuses. CCA occlusion experiments were performed under ketamine–xylazine (i.p. 100 to 10 mg/kg) anesthesia, while the whisker stimulation protocol and hypercapnic challenge was performed under mild ketamine-medetomidine (i.p. 30 to 0.1 mg/kg) sedation (Lecrux et al., 2017).

### Cisterna magna injection for drug delivery into the brain and i.p. drug administration

To block P2Y12R-mediated microglial actions, a P2Y12R antagonist, PSB-0739 (dissolved in 0.9% saline, 40 mg/kg in 5 μl volume; #3983; Bio-Techne Corp.) was injected into the cisterna magna 35 min before imaging, while vehicle (0.9% saline) injection was used as control. Cisterna magna injections were done under 1–1.5% isoflurane anesthesia. L-NAME, a nonselective NOS inhibitor (#0665; Tocris) was injected i.p. (30 mg/kg dissolved in 0.9% saline) 5 min before imaging.

### SPECT and PET imaging

SPECT and PET studies were carried out on mice anesthetized with 2% isoflurane (Apostolova et al., 2012). SPECT measurements were performed using the [99mTc]-HMPAO ligand (hexamethylpropylene amine oxime; Medi-Radiopharma). The acquisition started 3 min after the i.v. injection of the radiotracer via the tail vein (injected activity: 99.22 ± 9.33 MBq). The measurements were performed on a NanoSPECT/CT PLUS device (Mediso) equipped with multipinhole mouse collimators. Measurements were reconstructed with 0.25-mm isovoxels, and the results were quantified as units of radioactivity (MBq/ml). After SPECT acquisition, [^18^F]-FDG PET measurements were performed. PET acquisition started 20 min after i.v. [^18^F]-FDG injection (2-deoxy-2-(18F)fluoro-D-glucose, injected activity: 12.05 ± 1.93 MBq; Pozitron-Diagnosztika) with an acquisition time of 10 min using a microPET P4 (Concorde Microsystems). A maximum a posteriori algorithm was used to reconstruct the data with 0.3-mm isovoxels. After reconstruction, manual coregistration and atlas-based ROI measurements were done using VivoQuant software (InviCRO) in the cerebellum, cerebral cortex, and whole brain. For microglia-depleted and control groups, mean [^18^F]-FDG and [99mTc]-HMPAO standardized uptake values were analyzed by using two-way ANOVA followed by Sidak’s post hoc test (GraphPad Prism 7.0) and a permutation *t* test in R 3.5.1 (R Foundation for Statistical Computing).

### Whisker stimulation protocol

Whisker stimulation was performed manually and electromechanically (with a bending actuator, #PL112-PL140; PICMA; bender connected to a piezo amplifier, #E-650 Amplifier, Physik Instrumente). For manual stimulation, an earpick was used (at 4–5 Hz frequency) according to the following protocol: left whiskers were stimulated for 30 s, repeated 6 times, 60 s apart. During electromechanically controlled stimulation (5 Hz), whiskers were stimulated for 15 s, repeated 10 times with 40-s intervals. Stimulation-evoked CBF responses in the contralateral barrel cortex were recorded. CBF measurements were carried out under ketamine-medetomidine sedation (30 to 0.1 mg/kg dissolved in 0.9% saline, i.p.). All coupling experiments performed were time-matched from the time of anesthetic injection to ensure comparable results across different experiments.

### fUS

fUS acquisition was done with a 15-MHz probe of 128 elements (Vermon SA) connected to a prototype ultrafast research ultrasound scanner (hardware and software functionally analogous to the Iconeus One system; Iconeus). Recordings were performed through the skull while the animal was anesthetized with ketamine-medetomidine (i.p. 30 to 0.1 mg/kg). The head of the animal was shaved and fixed into a stereotactic frame. The probe was positioned using a built-in software based registration to the 3D Allen Brain Atlas (2015 Allen Institute for Brain Science, Allen Brain Atlas API, available from brain-map.org/api/index.html). Doppler images were obtained as described earlier (Tiran et al., 2017). 11 tilted planes were insonificating the medium at 5,500-Hz pulse repetition frequency to compute one compounded image every 2 ms. Of a block of 200 images, a Power Doppler image was obtained by removing the 60 first modes of SVD decomposition to extract the blood signal (Demene et al., 2015) from tissue clutter at a 2.5-Hz sampling rate. Acquisition started and ended with a 5-min baseline followed by 10 phases of 30-s manual stimulation of the whiskers (Ferrier et al., 2020) with 1 min of resting in between. A fourth-order polynomial detrending of the data was applied to remove drifts of baseline (Rabut et al., 2020).

### In vivo electrophysiology

Surgical procedures, microdrive construction, and implantation have been described previously (Kvitsiani et al., 2013). Briefly, custom-built microdrives with eight nichrome tetrodes (diameter, 12.7 µm; Sandvik) and a 50-µm core optic fiber (outer diameter, 65 ± 2 µm; Laser Components) were implanted into the right barrel cortex: anteroposterior, −1.4; mediolateral, 3.0; and dorsoventral, 0.75–2.0 mm. Although photostimulation was not applied here, the optic fiber is part of our typical drive design as it also provides mechanical support for the tetrodes. The microdrive contained a moveable shuttle, allowing more precise targeting. The custom-built microdrives were implanted under deep anesthesia. The stereotaxic surgery was followed by a 3-d-long resting period. Tetrodes were lowered (40–120 μm based on the estimated electrode positions and the presence of single units) between recording sessions to collect neuronal activity from different dorsoventral positions. The experiment was repeated two or three times, with a 2-d gap between sessions. Every session started with a 5-min recording without stimulation, defined as basal activity. Automated whisker stimulation epochs lasted for 15 s with 5-Hz frequency, followed by a 40-s-long interstimulus period. Stimulation was repeated 10 times. The entire protocol was repeated with the stimulator positioned close to the whiskers without touching them, to provide a sham stimulation condition that allowed us to exclude possible contaminations from electric noise from the stimulator circuit in our recordings. Next, manual whisker stimulation was applied (15-s stimulation with 40-s interstimulus period, repeated two times). Finally, changes in neuronal firing were measured during a 2-min-long hypercapnic challenge, by inhalation of a 10% CO_2_ containing air mixture (21.1% O_2_ and 68.9% N_2_) under normoxic conditions. Data acquisition was conducted with an Open Ephys (open source data acquisition system, hiv4) board, synchronized with the electromechanical whisker stimulator through a pulse generator (PulsePal 1102; Sanworks; Siegle et al., 2017). Data analysis was performed in Matlab R2016a (MathWorks). Spike sorting was carried out using MClust 3.5 (A.D. Redish). Only neurons with isolation distance >20 and L-ratio <0.15 (a cluster quality measure based on Mahalanobis-distance; Schmitzer-Torbert et al., 2005) were included.

### Primary microglial cell cultures

Primary cultures of astroglial cells were prepared from neonatal mouse brains, as described earlier (Kornyei et al., 2005). In brief, meninges were removed from postnatal day 0–2 whole brains and tissues were chopped. The tissue pieces were digested with 0.05% wt/vol trypsin and 0.5 mg/ml DNAse I (#T4549, #DN25; Sigma-Aldrich) in PBS for 10 min at RT. Cells were then plated onto plastic surfaces coated with poly-L-lysine (#P1524; Sigma-Aldrich) at a cell density of 3–4 × 10^5^ cell/cm^2^. The cultures were grown in minimal essential medium (#21090022; Thermo Fisher Scientific) supplemented with 10% FBS (#FB-1090; BioSera), 4 mM glutamine (#G3126; Sigma-Aldrich), and 40 μg/ml gentamycin (Sandoz). The culture medium was changed twice a week. For the hypoxia/hypercapnia experiments, the primary cultures were passaged and plated at 1.5 × 10^5^ cell/cm^2^ density into poly-L-lysine–coated 48-well plates and used within 96 h. Astrocytes no older than 6–8 days in vitro were used. Primary microglia cells were isolated from astroglia/microglia mixed cultures derived from the whole brains of C57BL/6J newborn mouse pups. Microglia isolation was performed between days 21 and 28 of culture maintenance, by mild trypsinization (Saura et al., 2003). For the in vitro hypoxia and hypercapnia experiments, the isolated cells were seeded at 1.5 × 10^5^ cell/cm^2^ density into poly-L-lysine–coated 48-well plates and used within 48 h.

### Primary microglia cell cultures and calcium imaging

Primary microglia cells were isolated from astroglia/microglia mixed cultures (described above) derived from the whole brains of MicroDREADD^Dq^ newborn mouse pups. Half of the mixed cultures were treated repeatedly with 2 µM 4-hydroxy TMX (#H6278; Sigma-Aldrich) for 1 wk before microglia isolation, which was performed between days 21 and 28 of culture maintenance, by mild trypsinization (Saura et al., 2003). The cells were seeded onto poly-L-lysine–coated glass coverslips at cell density of 40,000 cell/cm^2^ and used for imaging within 2–3 days. Phase-contrast time-lapse images to assess microglia process motility were captured on a Zeiss Axiovert 200M microscope at 20× magnification (20× Plan-Neofluar Ph2 objective), with a frame rate of 0.2 fps. For calcium imaging, the cells were loaded with 1 µM Oregon Green 488 BAPTA-1 AM (#O6807; Invitrogen) or 5 µM Calbryte 590 AM (#20701; AAT Bioquest) dyes in the presence of 2,000× Pluronic F-127 (#P6866; Invitrogen) or 100× PowerLoad Concentrate (#P10020; Invitrogen) for 30 min at RT. During imaging, the cells were perfused with ACSF (125 mM NaCl, 2.5 mM KCl, 8 mM NaHCO_3_, 1 mM MgCl_2_, 2 mM CaCl_2_, 20 mM Hepes acid, and 10 mM glucose) with a flow rate of 1 ml/min at RT. In some experiments, no perfusion was used. The cells were treated by DREADD agonists CNO (1 µM or 100 nM; #6329; Bio-Techne Corp) or C21 (1 µM; #HB6124; HelloBio). In experiments related to Fig. S2, the cultures were repeatedly treated with 1 µM C21 for 1 min, 10 min apart, followed by a single application of 10 µM ATP. Calcium imaging was performed either on a Nikon A1R confocal laser-scanning system built on a Ti-E inverted microscope, at 60× magnification (60× Plan Apo VC WI objective, NA = 1.2), with a frame rate of 20 fps, or on a Nikon Ti2 microscope equipped with a CoolLed pE-4000 illumination system and a Hamamatsu ORCA-Flash 4.0 camera, at 40× magnification (40× Apo WI λS objective, NA = 1.25), with a frame rate of 2 fps. Signal extraction from the time-lapse series was computed in Fiji (ImageJ; National Institutes of Health [NIH]), and the data were analyzed on Clampfit (pClamp10 suite; Molecular Devices). Statistics were calculated with GraphPad Prism 8.4.3.

### Induction of hypercapnia in vivo

Hypercapnia was induced by inhalation of a 10% CO_2_-containing air mixture (21.1% O_2_ and 68.9% N2) for 2 min under normoxic conditions under mild ketamine-medetomidine (i.p. 30 to 0.1 mg/kg) sedation, followed by a 2-min-long posthypercapnic imaging period. In a group of control and microglia-depleted mice, before the hypercapnic challenge, 0.01 µg/g atipamezole (Revertor, 5 mg/ml; CP-Pharma) was administered i.p. to withdraw α-2-agonistic effects of medetomidine. 3–5 min were allowed to get the effect of atipamezole established, before recording baseline CBF and induction of hypercapnia.

### Acute slice hypercapnia experiment and cGMP immunolabeling

Mice were deeply anesthetized with isoflurane (*n* = 3) and decapitated, brains were removed, and 300-µm-thick horizontal hippocampal slices were cut on vibratome (VT1200S; Leica). Slices were placed into an interface-type incubation chamber that contained standard ACSF at 35°C that gradually cooled down to RT. Slices were preincubated for 20 min with 1 ml of modified ACSF (mACSF) containing 1 mM 3-isobutyl-1-methylxanthine, 10 µM BAY 73-6691 phosphodiesterase inhibitors (to avoid cGMP hydrolysis), and 0.2 mM L-arginine (the substrate of NOS). L-arginine (0.2 mM) alone had no effect on NOS activity and cGMP levels (Garthwaite et al., 1989). For selective blockade of the microglial P2Y12R, 2 µM PSB 0739 (in mACSF) was used. After preincubation in mACSF or mACSF + PSB, slices were gradually subjected to hypercapnia by elevating the CO_2_ level from 5% to 14.6% with bubbling. 200 µM SNP (NO donor) was used as a positive control. After 15-min hypercapnia, the slices were immediately fixed with ice-cold 4% paraformaldehyde for 48 h at 4°C. After washing with 0.1 M PB, slices were embedded to 4% agar and 50-µm-thick vibratome (VT1200S; Leica) sections were cut followed by an immunofluorescent labeling. Sections were incubated in the following primary antibody mixture: sheep anti-cGMP (1:4,000; (de Vente and Steinbusch, 1997), rat anti-CD13 (1:500; MCA2183EL; Bio-Rad), biotinylated tomato Lectin (1:500, B-1175; Vectorlabs), and rabbit anti-P2Y12R (1:2,000, 55043A; AnaSpec) diluted in PBS for 48 h at 4°C. After subsequent washes in PBS, sections were incubated in a mixture of corresponding secondary antibodies (all from Jackson ImmunoResearch): donkey anti-sheep Alexa Fluor 488 (1:500; 713-546-147), donkey anti-rat Alexa Fluor 647 (1:500; 712-606-153), streptavidin Dylight405 (1:500; 016-470-084), and donkey anti-rabbit Alexa Fluor 594 (1:500; 711-586-152) diluted in PBS. Sections were mounted onto glass slides, coverslipped with Slowfade Diamond antifade mountant (S36972; RI: 1.52; Invitrogen) and Menzel-Glaser coverslip (#1). All steps were performed below 4°C. Fluorescent images were acquired using a Nikon Eclipse Ti-E inverted microscope (Nikon Instruments Europe B.V.), with a Plan Apochromat VC 20× objective (NA 0.75) and an A1R laser confocal system; scanning was done in line serial mode, and pixel size was 0.31 µm. Image stacks were obtained with NIS-Elements AR. 3-Isobutyl-1-methylxanthine), BAY 73-6691 (1-(2-chlorophenyl)-6-[(2*R*)-3,3,3-trifluoro-2-methylpropyl]-1,5-dihydro-4*H*-pyrazolo[3,4-*d*]pyrimidine-4-one), L-arginine, and SN) were purchased from Sigma-Aldrich and PSB0739 from Tocris.

### Simultaneous measurement of CBF and brain pH during hypercapnia

Electrophysiological variables (DC potential, brain pH) and local CBF (by laser Doppler) were simultaneously monitored after craniotomy using ion-sensitive microelectrodes connected to a custom-made dual-channel high-input impedance electrometer (including AD549LH; Analog Devices) via Ag/AgCl leads and associated filter modules (NL106 and NL125; NeuroLog System; Digitimer). Ion-sensitive microelectrodes were prepared according to Voipio and Kaila (1993). In each experiment, a pH-sensitive microelectrode was lowered into the cortex with a micromanipulator, together with another glass capillary microelectrode (tip diameter, 20 μm) filled with saline to serve as reference. The tips of the two electrodes were positioned as near as possible. The reference electrode acquired slow cortical or DC potential. An Ag/AgCl electrode was implanted under the skin of the animal’s neck to be used as common ground. The voltage signal recorded by the reference electrode was subtracted from that of the pH-sensitive microelectrode by dedicated differential amplifiers and associated filter modules (NL106 and NL125; NeuroLog System; Digitimer), which yielded potential variations related to changes in H^+^ ion concentration. The recorded signals were then forwarded to an analogue-to-digital converter (MP 150; Biopac Systems). Electric signals were continuously acquired at a sampling frequency of 1 kHz using the software AcqKnowledge 4.2.0 (Biopac Systems). Extracellular pH changes were expressed in millivolts to be translated into pH units offline, using least squares linear regression. The laser-Doppler flow signal was digitized and displayed together with the DC potential and pH signals (MP 150 and AcqKnowledge 4.2.0; Biopac Systems). Surgical preparations were done under 1.5–2% isoflurane, and pH and laser-Doppler flow measurements were performed under medetomidine anesthesia (initiation: i.p. 0.5 mg/kg, repeated 5 min later for maintenance) in a Faraday cage. After 15-min baseline acquisition, 2 min hypercapnia was imposed by CO_2_-enriched gas inhalation (9.7% CO_2_, 21% O_2_ in N_2_; Messer) at spontaneous respiration, which was repeated after a 5-min resting period.

### Primary endothelial cells

Primary endothelial cultures were prepared from 6–8-wk-old C57BL/6J mouse brains as described earlier (Deli et al., 2003), now performed with modifications (Lenart et al., 2015). In brief, mouse forebrains were collected to PBS, and the meninges were removed using sterile chromatography paper. The tissue was cut into small pieces by scalpels and was enzymatically digested in a mixture of Collagenase II (CLS2, 1 mg/ml; #C6885; Sigma-Aldrich) and DNase I (0.025 mg/ml; ∼50 U, #D4513; Sigma-Aldrich) in DMEM-F12 (#10-103-CV; Corning) for 55 min at 37°C. Using a 20% BSA (#A7906; Sigma-Aldrich, in DMEM-F12) gradient (1,000 *g*, 20 min; three times), microvessels were separated from the myelin. The collected microvessels were further digested using a mixture of Collagenase/Dispase (1 mg/ml; #11097113001; Sigma-Aldrich) and DNase I (0.038 mg/ml; ∼75 U; #D4513; Sigma-Aldrich) for 35 min at 37°C. Digested cerebral microvessels were washed three times with DMEM-F12, then seeded to plates coated with Collagen type I (#354236; Corning). During the first 4 d, puromycin (Perriere et al., 2005; Perriere et al., 2007; 4 µg/ml, #P7255; Sigma-Aldrich) selection was applied in the primary medium (15% PDS [#60-00-850; First Link] for seeding, 10% for cultivation, 1 ng/ml bFGF [#F0291; Sigma-Aldrich], 100 µg/ml heparin [#H3149; Sigma-Aldrich], 100× ITS [#41400045; Gibco], and 4 µg/ml puromycin in DMEM-F12) to selectively eliminate non–P-gp-expressing cells. After reaching confluency in 5–6 d, the cells were passaged to 48-well plates coated with Collagen type IV (100 µg/ml; #C5533; Sigma-Aldrich) and fibronectin (25 µg/ml; #F1141; Sigma-Aldrich) at a cell density of 15,000 cells/well and used for in vitro hypoxia or hypercapnia experiments in Passage 1.

### In vitro hypoxia and hypercapnia

Cytation 5 Cell Imaging Multi-Mode Reader (BioTek) equipped with O_2_/CO_2_ gas controllers was used to maintain 1% O_2_/5% CO_2_/94% N_2_ (hypoxia) or 15% CO_2_/85% air (hypercapnia) levels at 37°C. Endothelial or astroglial cells grown in 48-well plates to confluency or microglia were placed into the reading chamber of the instrument for 5 min (hypercapnia) or 10 min (hypoxia), after taking the lids off. To avoid medium change–induced release events, cell culture medium was replaced with 400 µl complete fresh medium 16 h before the onset of the experiments. To follow the build-up of hypoxia at the cellular level, some cultures were loaded with 5 µM Image-iT Green Hypoxia Reagent (#I14834; Invitrogen) for 30 min at 37°C. The Hypoxia Reagent begins to fluoresce when atmospheric oxygen levels drop below 5%. Fluorescent images taken with Cytation5 (10× magnification) at 0/10 min were analyzed with Fiji software (v1.53; NIH), measuring mean gray values in 10 × 10-pixel ROIs of *n* = 50 individual cells from three independent experiments. Changes in medium pH during hypercapnia were measured by Phenol Red absorbance at 415 and 560 nm using the Cytation 5 Multi-Mode Reader (BioTek; Michl et al., 2019). Measurements were taken from 400 μl complete cell culture media in 48-well plates at 37 °C (*n* = 10). The ratios of the 415- and 560-nm peaks were analyzed against a calibration curve obtained from 10 mg/liter phenol red and 10% FBS containing PBS formulations at different pH, in the range of pH 5.5–8. Changes in the intracellular pH during hypercapnia were determined by fluorescence intensity readings of glial cells labeled with pHrodo Green AM (#P35373; Invitrogen) on a Cytation 5 Multi-Mode Reader (*n* = 4). Intracellular pH calibration was performed by incubating the pHrodo Green AM–labeled cells in ACSF set to different pH values of pH 5.5–7.5 and supplemented with 10 µM nigericin and 10 µM valinomycin (#431; #3373; Bio-Techne Corp.) for 5 min.

### Quantification of nucleotides and nucleoside

Released concentrations of adenine nucleotides (ATP, ADP, and AMP) and adenosine (Ado) from culture media and tissue homogenates were determined using HPLC by Shimadzu LC-20 AD Analytical System using UV detection (Agilent 1100 VW set at 253 nm). Concentrations were calculated by a two-point calibration curve using internal standard method. The data (*n* = 4 or 5 in each group) are expressed as nanomoles per milliliter. Briefly, the medium (400 μl) was transferred into a cold Eppendorf tube that contained 50 μl of 0.1 M perchloric acid with 10 µM theophylline (as an internal standard) solution, then samples were centrifuged (at 3,510 *g* for 10 min at 0–4°C) and the supernatants were kept at −20°C until analysis. The weighed frozen tissue was homogenized in the same solution as the media, and the precipitated protein content was removed by centrifugation at 3,510 *g* for 10 min at 4°C. The pellet was saved for protein measurement according to Lowry et al. (1951). A 4-M K_2_HPO_4_ solution was used to neutralize the supernatant, and the centrifugation step was repeated. The extracted purines were kept at −20°C until analysis. Online solid phase extraction coupled to the column-switching technique was applied to quantification of the nucleotide content of samples. HPLC separation was performed by Shimadzu LC-20 AD Analytical System using UV (Agilent 1100 VW set at 253 nm) detection. The phenyl-hexyl packed (7.5 × 2.1-mm) column was used for online sample enrichment and the separation was completed by coupling the analytical C-18 (150 × 2.1-mm) column. The flow rate of the mobile phases (phase A, 10 mM potassium phosphate buffer with 0.25 mM EDTA; phase B contained additional components such as 0.45 mM octane sulphonyl acid sodium salt, 8% acetonitrile [vol/vol], and 2% methanol [vol/vol], pH 5.55) was 350 or 450 μl/min, respectively, in a step gradient application (Baranyi et al., 2006). The sample enrichment flow rate of buffer A was 300 μl/min during 4 min, and the total runtime was 55 min. Concentrations of the homogenates were calculated by a two-point calibration curve using internal standard method. The data (*n* = 4 or 5 in each group) are expressed as picomoles per milligrams protein or nanomoles per milligram.

### Blood gas analysis

Arterial blood from the femoral artery under ketamine-medetomidine anesthesia (30 to 0.1 mg/kg ± 0.1 μg/g atipamezole) was sampled to glass capillaries and measured with a blood gas analyzer (ABL90 FLEX PLUS; Radiometer Medical) to determine arterial blood gas tensions (pO_2_, pCO_2_) and pH.

### Repeated, transient CCAo

Transient, repeated unilateral CCAo was performed to induce hypoperfusion without causing ischemia or cellular injury to the brain. The CCA was temporarily pulled away with a silk suture for 5 min, followed by a 5-min-long reperfusion period. The protocol consisted of repeating these steps three times (3× CCAo) on anesthetized (ketamine–xylazine, i.p. 100 to 10 mg/kg dissolved in 0.9% saline) mice. During CBF measurements, the core temperature of mice was maintained at 37 ± 0.5°C using a homeothermic blanket.

### Elimination of microglia or PVMs

C57BL/6J mice were fed a chow diet containing the CSF1R inhibitor, PLX5622 (1,200 mg PLX5622 in 1 kg chow; Plexxikon) to eliminate microglia from the brain (Szalay et al., 2016), or with control diet for 3 wk. PVMs were depleted by a single dose of clodronate-containing liposomes (70 µg/mouse in 10-μl volume; #F70101C-N-2; FormuMax Scientific) injected into the left ventricle (ICV) as described earlier (Faraco et al., 2016). 3 d later, at maximal efficacy of depletion, LSCI was carried out.

### Quantitative analysis

All quantitative analyses were done in a blinded manner. For the measurements of microglial process coverage of endothelial surface or pericytes, microglial process coverage was measured on confocal Z-stacks acquired with a step size of 300 nm. On single-channel images, lectin-positive vessels were selected randomly. The surface of these vessels was calculated by measuring their circumference on every section multiplied by section thickness. The length of microglial process contacts was measured likewise. Continuous capillary segments (<6 µm) were also randomly chosen, and the presence of microglial process contacts was examined. All labeled and identified pericytes (PDGFRβ-positive) were counted when the whole cell body was located within the Z-stack. 3D reconstruction of CLSM and two-photon imaging stacks was performed using the IMOD software package (Kremer et al., 1996). TOM20 fluorescent intensity profiles were analyzed using a semiautomatic method (Fig. S1 d). Confocal stacks with triple immunofluorescent labeling (P2Y12R, TOM20, and Lectin) were collected. The image planes containing the largest diameter of longitudinal or cross-cut vessels were used to trace the outer membrane of endothelial cells based on the Lectin labeling. This contour was then expanded and narrowed by 0.5 µm to get an extracellular and an intracellular line, respectively. The intensity of fluorescent labeling was analyzed along these lines (TOM20 intensity along the intracellular, P2Y12R labeling along the extracellular line). After normalizing and scaling, microglial contacts were identified along the membrane of the endothelial cell, where microglial fluorescent intensity was over 20% of the maximal value, for at least along a 500-nm-long continuous segment. Then the contact area was extended 500 nm on both sides, and TOM20 fluorescent intensity within these areas was measured for contact value. TOM20 fluorescent intensity outside these identified contacts was considered noncontact.

For the analysis of GFAP^+^ astroglial cell body contact frequency, CLSM stacks with double immunofluorescent labeling (GFAP and P2Y12R) were acquired from mouse cerebral cortex. All labeled and identified astrocytes were counted when the whole cell body was located within the Z-stack. To assess microglia process motility, baseline (28 min) and after 3× CCAo (49 min) two-photon image sequences were exported from MES software v.5.3560 (Femtonics) and analyzed using Fiji (version 2.0.0; NIH). The acquired hyperstacks were motion corrected using the StackReg plugin, then individual perivascular microglia processes (30 processes/image/plane) were tracked using the Manual Tracking plugin of Fiji. Based on the obtained XYZ coordinates, process motility speed was calculated. To study the effects of 3× CCAo on microglial morphology, 3–3 C57BL/6J mice were randomized into two groups: CCAo or sham surgery. 24 h after 3× CCAo, mice were transcardially perfused and processed for automated microglial morphology analysis. In both cases, 100-µm-thick sections with microglia (Iba1) and cell nuclei (DAPI) labeling were imaged with CLSM (0.2 µm/pixel, Z-step of 0.4 µm). Obtained confocal stacks were processed with the Microglia Morphology Quantification Tool (Heindl et al., 2018). For LSCI recordings, venous sinuses were excluded from the analysis. LSCI generates relative perfusion values (arbitrary units); therefore, CBF was expressed as percentage change over baseline values in the 3× CCAo experiments. For the CCA occlusion experiments, a 1-min long period (typically 250 datapoints), recorded at the beginning of the imaging session, was averaged and considered as baseline. Then, every occlusion and reperfusion event was normalized to baseline. To assess the plasticity of the cerebrovasculature in response to repeated hypoperfusion, normalized occlusion or reperfusion events were averaged and compared between experimental groups. To demonstrate the CBF kinetics of individual animals, every 20th image was extracted, and CBF values were presented on a scatter plot (Fig. S4 b). Quantification of P2Y12R immunostaining in control and microglia-depleted tissues or P2Y12R and CD206 immunostaining in control and clodronate-treated mice were performed in at least three randomly selected fields of view within the cortex on three different coronal planes in each mouse. Data obtained from every mouse brain were averaged and compared between experimental groups.

To investigate microglial actions on hypercapnic vasodilation, two-photon image sequences were exported from the MESc software v.3.5.6.9395 (Femtonics). After motion correction using the StackReg plugin of Fiji, the extent of vasodilation was measured and expressed as percentage of baseline at maximal vasodilation using Fiji. Obtained data were averaged and compared between control and microglia-depleted or between CX3CR1^GFP/+^ and CX3CR1^GFP/+^ × P2Y12R KO mice. The hypercapnia-evoked CBF responses (2 min long) were normalized to the baseline, then maximum values of individual responses were averaged per animal and compared between control and microglia-depleted groups. For the analysis of cGMP fluorescence, a systematic random sampling of parenchymal vessels based on Lectin staining was performed. These vessel segments were numbered, and their lumen diameter was measured. This was followed by masking the CD13^+^ profiles of these vessels, and automated cGMP intensity measurement was performed within these masks. Measurements were done using ImageJ software. Concentrations of released cellular purine nucleotides in response to hypoxia or hypercapnia were calculated by a two-point calibration curve, using internal standard method. The obtained values were averaged and compared with baseline ones.

For neurovascular coupling experiments (manual and electromechanical whisker stimulations) the stimulus-evoked responses were normalized to baseline and were expressed as CBF increase (% change). The evoked CBF responses were averaged per mouse. The magnitude of evoked CBF responses was compared between experimental groups. Before and after L-NAME injection, whiskers were stimulated, and the difference between the two sets of stimulations was analyzed and compared between the experimental groups. GCaMP6s signals of individual neurons were collected with MESc software v.3.5.6.9395 (Femtonics) and imported into the MES software v.5.3560 (Femtonics) curve analysis module. The individual cellular [Ca^2+^]_i_ traces were normalized to the baseline GCaMP6s signal, and data were expressed as relative fluorescence intensity change (Δ*F*/*F*). Then area under the curve (AUC) was calculated for each response, and AUC values were compared between experimental groups. During electrophysiological assessment, the baseline frequency of individual units was determined by averaging a 5-min-long period at the beginning of registration, when whisker stimulation was not applied. Only those units were selected for further analysis, which responded to electromechanical stimulations. Then the stimulus-evoked responses were corrected to baseline frequencies (called as baseline-corrected response frequency), and the magnitude of responses was compared between experimental groups. Signal extraction from the microglial [Ca^2+^]_i_ time-lapse series in response to ATP treatment was computed in Fiji. In brief, the mean fluorescence intensity values were determined in 10 × 10-µm ROIs (each representing an individual cell) and were used to calculate d*F*/*F* values (d*F*/*F* = [*F* − *F*0]/*F*0, where *F*0 is the average baseline fluorescence in a 300-s time window before drug application and *F* is the background-corrected fluorescence intensity value at a given time point). The data were further analyzed with Clampfit software (pClamp10 suite; Molecular Devices), manually determining the peak time and amplitude parameters and using the built-in functions to calculate peak amplitude, half width, and peak area.

### Statistical analysis

Animals were randomized for in vivo experiments using GraphPad Random Number Generator. Sample size was determined by a priori power calculation using G*, Power 3.1.9.2 with mean differences and 20–25% SDs based on pilot studies (power 80%, α = 0.05). Data were analyzed by GraphPad Prism 7.0 software, unless stated otherwise. Data were assessed for normal distribution using the D’Agostino–Pearson normality test or the Shapiro–Wilk *W* test to determine parametric or nonparametric analysis. For comparing two or more groups with normal distribution, unpaired *t* test with Welch’s correction and either one-way ANOVA with Dunnett’s multiple comparison test or two-way ANOVA with Tukey’s or Sidak’s multiple comparison test was used. For unevenly distributed data, the Mann–Whitney *U* test and either one-way ANOVA with Dunnett’s multiple comparison test or two-way ANOVA with Tukey’s or Sidak’s multiple comparison test was used. Refer to the figure legends and the results section concerning the actual study design. All data needed to evaluate the conclusions in the paper are presented in the paper and in the supplementary material. Additional data related to this paper may be requested from the authors.

### Online supplemental material

Fig. S1 presents further details on cellular anatomic interactions of microglia with astrocytes, pericytes, endothelial cells, and immunohistochemical characterization of microglia in CX3CR1^tdTomato^ mice, supporting findings in Fig. 1; confirmation for the efficacy of microglia depletion; and associated HMPAO-SPECT and FDG-PET measurements in relation to Fig. 2. Fig. S2 shows characteristics of in vitro–recorded microglial [Ca^2+^]_i_ responses and in vivo morphology changes in response to chemogenetic activation by C21 in connection with data presented in Fig. 4. Fig. S3 depicts in vivo two-photon imaging examples of microglial process dynamics around small capillaries in response to hypercapnia. For characterization of the hypercapnic challenge both in vivo and in vitro, measured blood gas parameters, extra- and intracellular pH values are provided, complementing results in Figs. 5 and 6. Fig. S4 shows the characteristic blood flow changes during CCAo as measured with LSCI in selected ROIs placed over the territory of MCA in line with results of Figs. 7 and 8. Effects of hypercapnia or microglial P2Y12R blockade on cGMP fluorescent intensity changes are also presented. Fluorescent intensity changes of Hypoxia Green have also been validated during in vitro hypoxic conditions. Video 1 demonstrates microglial process dynamics in CX3CR1^tdTomato^ mice corresponding to Fig. 1 b. While Video 8 represents perfusion changes during CCAo, Video 2 demonstrates the neurovascular coupling response in control and microglia-depleted mice recorded with LSCI. Video 3 provides in vivo two-photon imaging examples of neuronal [Ca^2+^]_i_ responses during whisker stimulation recorded in control and microglia-depleted Thy1-GCaMP6s mice related to Fig. 3, f and g. Videos 4 and 5 display in vitro microglial [Ca^2+^]_i_ dynamics and process motility changes in response to treatment with the C21 DREADD agonist corresponding to the results in Fig. 4. Video 6 recorded in CX3CR1^GFP/+^ × P2Y12R KO and CX3CR1^GFP/+^ mice show vasodilation during hypercapnia depicted in Fig. 5 j. Video 7 displays microglial [Ca^2+^]_i_ changes during hypercapnia-induced vasodilation as measured in CX3CR1^CGaMP5g–tdTomato^ mice using in vivo two-photon imaging depicted in Fig. 5c. Video 8 shows the third occlusion-reperfusion period in microglia-depleted and control mice. Table S1 provides information on patient data and the processing of postmortem human brain tissues shown in Fig. 1.

## Supplementary Material

Table S1 lists patient data and processing of postmortem human brain tissues.
