# Supplementary material for: Microglia modulate blood flow, neurovascular coupling, and hypoperfusion via purinergic actions
Source: J Exp Med. 2022 Feb 24;219(3):e20211071. doi: 10.1084/jem.20211071 (PMC8932534; doi:10.1084/jem.20211071)
Supplement: Table S1 — lists patient data and processing of postmortem human brain tissues. [file JEM_20211071_TableS1.docx]

**Table S1.** Patient data and processing of post-mortem human brain tissues.

| **Subject** | **Code** | **Gender** | **Age**  **(years)** | **Health status** | **Comorbidities** | **Cause of death** | **Tissue sample type** |
| --- | --- | --- | --- | --- | --- | --- | --- |
| Control subject | SKO13 | female | 60 | normal | chronic bronchitis | respiratory arrest | free floating and paraffin sections |
| Control subject | SKO16 | male | 73 | normal | Unspec. atherosclerosis, pneumonia | respiratory arrest | free floating and paraffin sections |
| Control  subject | SKO20 | male | 27 | normal | unspec. jaundice, malignant pancreatic neoplasm. | pulmonary embolism | free floating sections |
